# Incidence of induced abortion in Malawi, 2015

**DOI:** 10.1371/journal.pone.0173639

**Published:** 2017-04-03

**Authors:** Chelsea B. Polis, Chisale Mhango, Jesse Philbin, Wanangwa Chimwaza, Effie Chipeta, Ausbert Msusa

**Affiliations:** 1 Guttmacher Institute, New York, New York, United States of America; 2 Centre for Reproductive Health, College of Medicine, University of Malawi, Blantyre, Malawi; University of Ottawa, CANADA

## Abstract

**Background:**

In Malawi, abortion is legal only if performed to save a woman’s life; other attempts to procure an abortion are punishable by 7–14 years imprisonment. Most induced abortions in Malawi are performed under unsafe conditions, contributing to Malawi’s high maternal mortality ratio. Malawians are currently debating whether to provide additional exceptions under which an abortion may be legally obtained. An estimated 67,300 induced abortions occurred in Malawi in 2009 (equivalent to 23 abortions per 1,000 women aged 15–44), but changes since 2009, including dramatic increases in contraceptive prevalence, may have impacted abortion rates.

**Methods:**

We conducted a nationally representative survey of health facilities to estimate the number of cases of post-abortion care, as well as a survey of knowledgeable informants to estimate the probability of needing and obtaining post-abortion care following induced abortion. These data were combined with national population and fertility data to determine current estimates of induced abortion and unintended pregnancy in Malawi using the Abortion Incidence Complications Methodology.

**Results:**

We estimate that approximately 141,044 (95% CI: 121,161–160,928) induced abortions occurred in Malawi in 2015, translating to a national rate of 38 abortions per 1,000 women aged 15–49 (95% CI: 32 to 43); which varied by geographical zone (range: 28–61). We estimate that 53% of pregnancies in Malawi are unintended, and that 30% of unintended pregnancies end in abortion. Given the challenges of estimating induced abortion, and the assumptions required for calculation, results should be viewed as approximate estimates, rather than exact measures.

**Conclusions:**

The estimated abortion rate in 2015 is higher than in 2009 (potentially due to methodological differences), but similar to recent estimates from nearby countries including Tanzania (36), Uganda (39), and regional estimates in Eastern and Southern Africa (34–35). Over half of pregnancies in Malawi are unintended. Our findings should inform ongoing efforts to reduce maternal morbidity and mortality and to improve public health in Malawi.

## Introduction

About 56 million women worldwide are estimated to have an induced abortion each year [1]. Contrary to popular belief, regions with restrictive abortion laws do not have lower abortion rates than those with liberal laws [1], but women in restrictive settings are more likely to experience morbidity and mortality stemming from unsafe abortion [2–4]. In addition to serious health risks, unsafe abortion poses high financial costs and service burdens on families and on the national health system [5;6]. In Africa, more than one in every seven pregnancies (15%) end in an induced abortion [1], and the majority of women of childbearing age in Africa live in countries with restrictive abortion laws [7]. Africa accounts for about 15% of all abortions in the world [1], but contributes 65% of all estimated abortion deaths in the world [2].

While the maternal mortality ratio (MMR) in developed countries in 2015 averaged 12 maternal deaths per 100,000 live births, the MMR averaged 239 in developing regions, and more than double this (546) in sub-Saharan Africa [8]. Malawi has one of the highest MMRs in the world, even higher than in the rest of Africa, at 574 maternal deaths per 100,000 live births in 2014 [9]. The probability that a 15 year old Malawian girl will eventually die from maternal mortality is 1 in 29 [8]. Due to its persistently high MMR, Malawi failed to meet the Millennium Development Goal to reduce the MMR by 75% between 1990 and 2015. According to a review of largely hospital-based studies, abortions are estimated to contribute between 6–18% of maternal deaths in Malawi [10]. Medication abortion (use of either mifepristone plus misoprostol or misoprostol alone to induce an abortion), could significantly reduce maternal mortality in low-resource settings [11;12]. However, while misoprostol is registered in Malawi for prevention and treatment of post-partum hemorrhage and for post-abortion care, it is not believed to be widely used for induced abortion.

Abortion is only legal in Malawi if performed to save a woman’s life, and attempts to procure an abortion are punishable by 7–14 years imprisonment [13]. The majority of terminations in Malawi are performed under unsafe conditions [14], and the stigmatized nature of the procedure often leads to delays or avoidance in seeking medical care for complications [15]. Among women who do reach a facility for post-abortion care (PAC), more than one in four have severe or moderate morbidity, and there were an estimated 387 deaths per 100,000 PAC complications in 2009 [16].

Malawians are currently debating whether to liberalize the abortion law by providing more exceptions under which an abortion could be legally obtained. A Special Commission was established to investigate the issue, conduct national consultations, and make recommendations in a draft bill for Parliament to consider [17]. As part of this process, policymakers need reliable, up-to-date evidence on the magnitude and impact of abortion in Malawi. A 2009 study estimated a total of 67,300 induced abortions (range 48,600–86,000) in Malawi that year, equivalent to a rate of 23 abortions per 1,000 women aged 15–44 [18]. Between 2010 and 2015, the contraceptive prevalence rate in Malawi increased from 46% to 59% [19]. During this same period, the percent of adolescents aged 15–19 who had begun childbearing rose slightly from 25.6% to 29%.[19] Changes such as these could impact the abortion rate, rendering the 2009 estimates potentially outdated. We undertook a study to determine current estimates of induced abortion in Malawi.

## Methods

### Overview

We used an indirect estimation approach called the Abortion Incidence Complications Method (AICM) [20], which has been used in over 20 countries, including Malawi in 2009 [18]. It requires two surveys: a Health Facilities Survey (HFS) conducted in facilities with the potential to treat abortion complications, and a Knowledgeable Informants Survey (KIS), conducted among individuals knowledgeable about abortion in the country.

Women undergoing induced abortion have three potential outcomes: experiencing no complications, experiencing complications but not obtaining treatment in a health facility, or experiencing complications and obtaining treatment in a health facility. Data from the HFS is used to estimate the latter. KIS respondents estimate the distribution of abortions by provider type, the probability of experiencing complications by provider type, and the probability of obtaining PAC in a facility. Given differentials in women’s access to abortion and to PAC, these probabilities are estimated separately for four sub-groups: rural poor and non-poor women, and urban poor and non-poor women. This information is used to calculate a multiplier which represents, for each induced abortion complication treated, how many induced abortions occurred for which treatment was either not required or not obtained. Applying the multiplier (calculated from the KIS) to the estimated number of induced abortions with treated complications (calculated from the HFS) yields an estimate of all induced abortions in the country.

Fieldwork occurred during October-December 2015, led by the Centre for Reproductive Health at the College of Medicine, University of Malawi, with technical support from the Guttmacher Institute. Twelve interviewers with previous experience administering surveys related to reproductive health conducted HFS and KIS interviews, supervised by three MPH-level interviewers who also administered several KIS interviews. All of these interviewers were in turn supervised by a Project Coordinator. A week-long training for all interviewers, supervisors, and study staff was held in Blantyre just prior to fieldwork initiation. We pilot tested questionnaires in a small number of interviews with respondents outside of our sample.

Ethical approval to conduct the study was obtained from Guttmacher’s Institutional Review Board and the Research and Ethics Committee of the College of Medicine. We also obtained a letter of support from the Malawi Ministry of Health, Department of Planning and Policy Development. Respondents in both surveys provided written informed consent, and interviews were conducted in English or Chichewa (the vernacular of Malawi), according to the respondent’s preference. We did not provide incentives for participation in either survey.

### HFS sampling and fieldwork

We obtained a list of 977 facilities from the 2013–14 Malawi Service Provision Assessment [21], which represented 92% of all known health facilities in the country (Table 1). We excluded 49 facilities too specialized to provide PAC (e.g., dental offices, prison clinics, podiatry clinics), plus 47 dispensaries and 20 health posts, since PAC services are not offered at this level of health care facility in Malawi. Among 861 remaining facilities, we selected a nationally representative sample using stratified random sampling by facility level (central hospital, district hospital, rural/community hospital, other hospital, health centre, clinic, maternity unit), administrative zone of the Ministry of Health (North, Central-East, Central-West, Southeast, and Southwest) and ownership type (government, non-governmental organization [NGO], or private). We included all hospitals and maternity units, and randomly sampled 40% of health centers and 12% of clinics, for a total of 334 sampled facilities.

**Table 1:** Number of facilities sampled, Health Facilities Survey, Malawi 2015.

Facility type	# facilities in 2013–4 MSPA	# facilities possibly providing PAC	% of facilities sampled	# facilities sampled*
Central hospital	4	4	100%	4
District hospital	24	24	100%	24
Rural/community hospital	41	41	100%	41
Other hospital	47	40	100%	40
Health centre	473	467	40%	187
Clinics	317	281	12%	34
Maternity unit	4	4	100%	4
Dispensaries	47	0	n/a	n/a
Health Posts	20	0	n/a	n/a
**TOTAL**	977	861		334

* To determine the number of health centers and clinics to be sampled within each geographical zone, we determined the proportion of all health centers and clinics represented in each geographical zone. We then applied the relevant proportion to the total number of health centers (187) or clinics (34) we planned to sample. To determine the number of health centers and clinics to be sampled within each geographical zone and by ownership, we determined the proportion of health centers and clinics within each geographical zone that were within each ownership category (public, NGO, and private). We then applied the relevant proportion for each type of health facility (health centre or clinic) and zone to the total number of health centers or clinics that we planned to sample in each geographical zone.

To minimize refusals, the Malawian Ministry of Health sent letters introducing the study and noting an upcoming interview request to the 334 sampled facilities in advance of data collection. Interviewers used a standardized questionnaire to conduct a face-to-face interview with a senior staff member knowledgeable about the facility’s provision of abortion services; generally a nurse-midwife (68%) or other clinician (27%).

### KIS sampling and fieldwork

In consultation with zonal officers and reproductive health experts in Malawi, we developed a purposive sample of 125 potential knowledgeable informants; 25 per zone, representing 27 of Malawi’s 28 districts (the small Lake Malawi island district of Likoma was excluded due to accessibility issues). Trained interviewers successfully interviewed all 125 invited respondents, who held a range of professions, including formal sector medical providers (45%), community health workers (19%), NGO employees (11%), village health committee members (6%), traditional birth attendants (5%), or other professions (14%). Respondents were balanced by gender (52% male and 48% female). Almost half (46%) worked in the public sector, with 29% in NGOs, 12% in the private sector, 5% affiliated with a Christian Health Association of Malawi (CHAM) facility, and 8% in other sectors (for example, a traditional leader or member of a community health committee). The 2009 Malawi AICM [18] noted concern about potential underrepresentation of informants familiar with the situation of abortion in rural areas, and recommended that future studies identify informants with recent rural experience. Thus, we paid particular attention to this factor. While 16% of our respondents worked in urban areas, 42% worked in rural areas and another 42% worked in both urban and rural areas. Interviewers determined that the respondent knew about abortion in rural areas very well or moderately well in 91% of interviews. In 10 of 125 KIS interviews, interviewers noted concern about respondent uncertainty in responses to key questions. We excluded these 10 respondents from analysis, after confirming that they were similar to the remaining sample of KIS respondents in terms of location, gender, age, or years in profession.

### Analysis

Out of 334 HFS facilities sampled, we successfully interviewed 294 facilities (88% of sampled facilities) (Table 2). Nine were either closed, not feasible to access, or had merged with another clinic in our sampling frame. An additional 31 facilities refused to participate or did not have the necessary staff member available to give an interview. Of these, 3 refused interviews for unknown reasons and 6 did not have a staff member available to give the interview. Twenty-two refusals (including 11 clinics, 7 health centers, and 4 “other” hospitals) occurred because that facility did not offer PAC services. Of the 294 interviewed facilities, 202 reported providing PAC, with greater likelihood in higher-tier facilities (i.e., hospitals, 79–100%) than lower-tier facilities (i.e., clinics or health centers, 50–60%) or maternity units (25%) (Table 2).

**Table 2:** Facilities sampled, interviewed, and providing PAC, with estimated national PAC caseloads, Health Facilities Survey, Malawi 2015.

Facility type	# facilities sampled*	# sampled facilities completing interviews	% sampled facilities completing interviews	# interviewed facilities providing PAC	% interviewed facilities providing PAC	Estimated annual PAC caseload (weighted) **	% of caseload by facility type
Central hospital	4	4	100%	4	100%	4,398	6%
District hospital	24	24	100%	23	96%	25,674	35%
Rural/community hospital	41	39	95%	39	100%	7,494	10%
Other hospital	40	28	70%	22	79%	5,114	7%
Health centre	187	174	93%	103	60%	16,520	22%
Clinics	34	21	62%	10	50%	15,038	20%
Maternity unit	4	4	100%	1	25%	90	<1%
**TOTAL**	334	294	88%	202	69%	74,328	100%

* Out of 334 HFS facilities sampled: 4 were not feasible to access; 1 had merged with another clinic in our sampling frame; 4 were permanently closed; 6 did not have a provider available to give an interview during the fieldworkers’ visit to the area; 22 refused to provide an interview because they didn’t offer PAC; and 3 refused an interview for some other reason.

** Weighted by the inverse of the product of the sampling fraction and the response rate for each facility type to yield results that were nationally representative.

From these 202 facilities, we obtained estimates of the number of PAC patients (inpatient or outpatient) treated for either miscarriage or induced abortion (as the two are often clinically indistinguishable, and may not be reported separately for fear of legal sanctions) in an average month and in the past month. We averaged these two figures and multiplied by 12 to estimate the total number of PAC cases in interviewed, PAC-providing facilities in 2015. We then weighted those values by the inverse of the product of the sampling fraction and response rate for each facility type to provide nationally representative estimates of PAC caseloads by facility type. We assumed that the proportion, by facility type and ownership, of PAC-providing facilities in the sample was similar to the proportion of PAC-providing facilities in the country, and adjusted the sampling frame accordingly.

To avoid double-counting PAC patients who may have been treated at one facility and then referred for additional care to a higher-level facility, we subtracted cases believed to be referrals. In order to do this, we asked each facility how many PAC patients they treated and then referred elsewhere. In the absence of a reliable referral follow-up rate estimate in Malawi, we assumed that 75% of referred patients followed up, as was done in a recent AICM study in Tanzania [22], based on data from a prospective study of follow-up for obstetric complications (specifically intrauterine fetal death) in a rural district of Tanzania [23]. Typically in Malawi, health centers, clinics, maternity units, and rural/community hospitals refer to district and CHAM hospitals, which in turn refer to central and top-tier private hospitals. In each zone, we subtracted 75% of referrals out of health centers, clinics, maternity units, and rural/community hospitals from the number of PAC patients treated at district hospitals and CHAM hospitals; and also subtracted 75% of referrals from district and CHAM hospitals from the number of PAC patients treated at central hospitals and high-level private hospitals (Table 3). This correction for double-counting reduced the number of PAC cases by 10%.

**Table 3:** Post-Abortion Care (PAC) cases in Malawi, Health Facilities Survey, Malawi 2015.

Zone	(A) Estimated PAC caseload (weighted), 95% CI	(B) Estimated # of double-counted referrals (weighted)	(C) Estimated # late miscarriages treated for PAC	(A-(B+C)) Estimated # induced abortions receiving PAC (weighted), adjusted 95% CI*	Induced abortion complications treatment rate**	% of caseload of induced abortions by zone	% of caseload of induced abortions by region***
**National**	**74,328 (67,063–81,593)**	**7,305**	**15,328**	**51,693 (44,429–58,959)**	**14 (12–16)**		
North	15,473 (14,561–16,385)	1,180	2,083	12,210 (11,298–13,123)	24 (22–26)	23.6%	23.6%
Central-West	18,360 (16,322–20,398)	2,595	4,020	11,745 (9,707–13,784)	12 (10–14)	22.7%	39.2%
Central-East	12,432 (10,977–13,887)	1,194	2,723	8,515 (7,060–9,970)	14 (12–17)	16.5%	
Southwest	16,292 (14,466–18,117)	826	3,898	11,567 (9,742–13,393)	13 (11–15)	22.4%	37.2%
Southeast	11,771 (10,737–12,805)	1,511	2,604	7,656 (6,622–8,690)	10 (9–12)	14.8%	

* Adjusted 95% CIs were calculated by subtracting referrals and late miscarriages from the upper and lower bounds of the 95% CIs for the estimated number of all PAC cases.

**Refers to the number of PAC cases stemming from induced abortions per 1,000 women ages 15–49

***Malawi is divided into three administrative regions: Northern, Central, and Southern. The Ministry of Health divides the country into five administrative zones: the Southern region is comprised of the Southwest and Southeast zone, the Central region is comprised of the Central-West and Central-East zone, and the Northern region is comprised of the North zone.

Next, we subtracted PAC cases likely due to miscarriage, in order to estimate PAC cases stemming specifically from induced abortion. The number of pregnancies ending in late miscarriage (between 13 and 21 weeks gestation) is estimated to equal 3.41% of all live births [24;25], and we assumed that only later miscarriages likely require PAC. We estimated the total number of live births by multiplying, separately for urban and rural women, age-specific fertility rates with the number of women in the population by age groups [19], and then adding these together for the total number of births among women aged 15–49. Since not all late miscarriages require care in a facility, we asked KIS respondents to estimate, separately for urban and rural women, the proportion of late miscarriages treated in a health facility, and calculated the median of their responses (90% among urban women, 70% among rural women). Applying these proportions to the estimated number of late miscarriages represents the total number of PAC cases likely due to miscarriage (Table 3).

To generate the multiplier, we multiplied the estimated probabilities of experiencing complications of induced abortion according to provider type from KIS data against the probabilities of receiving treatment for complications, among urban poor, urban non-poor, rural poor, and rural non-poor women, also from KIS data. This yields the estimated proportion of abortions with complications that receive treatment among these four groups. Separately for each health zone, these estimates are combined into a single weighted proportion based on the population distribution of the four groups [26], defining “poor” as belonging to a household with below median wealth and assets, according to the Malawi 2010 Demographic and Health Survey.

We used Taylor linearized variance estimation, using a finite population correction factor, to calculate 95% confidence intervals (CIs) around PAC caseloads, reflective of the complex survey design. We used a scaling factor (the average of the variances from strata with multiple sampling units) for strata containing a single population sampling unit. Since we subtracted likely referrals and late miscarriages from the PAC caseloads estimate, we adjusted the 95% CIs accordingly, by subtracting the same amount from both the upper and lower bounds. We applied multipliers to the estimated number of induced abortions requiring and receiving treatment (and to the adjusted upper and lower bounds), to produce an estimate (and associated 95% CI) for the total number of induced abortions in Malawi in 2015 (Table 3). Mathematical expressions for our calculations are provided in the Appendix (**Text A in** S1 File).

Finally, we used these abortion estimates to calculate the number and rate of unintended pregnancies nationally and by zone, by building upon information on unplanned births from DHS to estimate rates, outcomes, and intention status for all pregnancies (versus only those ending in a live birth). We used information about the planning status of births (collected retrospectively, pertaining to a woman’s reported fertility desires at the time she became pregnant) in the past five years from the 2010 DHS to obtain the percentage of live births that were unplanned. We assume that all abortions were the result of unintended pregnancies. Miscarriages of any gestational age were estimated as 20% of live births plus 10% of abortions [25]. The estimate for unintended pregnancies, then, is the sum of unintended births, abortions, and miscarriages of pregnancies believed to be unintended, i.e. 20% of unintended live births plus 10% of abortions. Likewise, the estimated number of intended pregnancies consists of intended births plus the estimate of miscarriages of intended pregnancies, which is equal to 20% of intended births. From the estimated number of unintended pregnancies, we calculated an unintended pregnancy rate and the percentage of pregnancies in Malawi and in each zone that was unintended.

## Results

### PAC caseloads

We estimated that health facilities treated a total of 74,328 (95% CI: 67,063 to 81,593) PAC cases (miscarriages or induced abortions, and including potential double-counting) in Malawi in 2015 (Table 2). After excluding an estimated 7,305 double-counted referral visits and 15,328 PAC cases due to late miscarriages, we estimated that 51,693 (95% CI: 44,429 to 58,959) induced abortions had complications and received PAC in Malawi in 2015 (Table 3). This translates to a rate of 14 PAC cases due to induced abortion per 1,000 women ages 15–49. The proportion of the national caseload of women treated in facilities for complications of induced abortion, by zone and region, are provided in Table 3; with the highest proportion in the North zone (23.6%) and the lowest in the Southeast zone (14.8%).

### Complication and treatment probabilities for induced abortion

**Fig 1:**
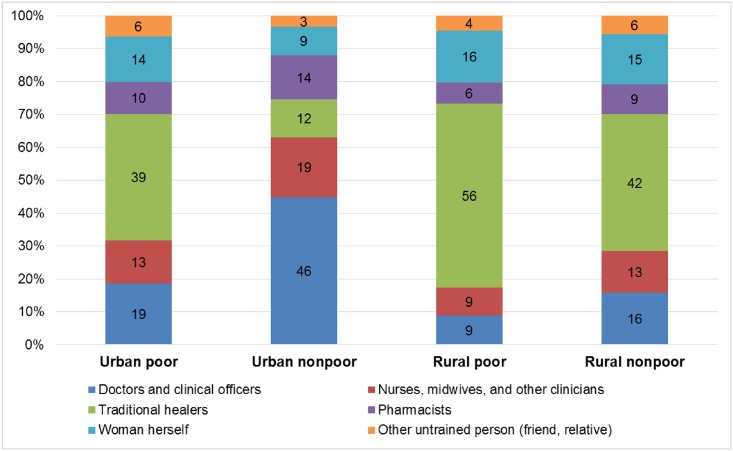
Abortion procurement by provider type, according to women’s sociodemographic characteristics, Knowledgeable Informant Survey, Malawi 2015.

**Fig 2:**
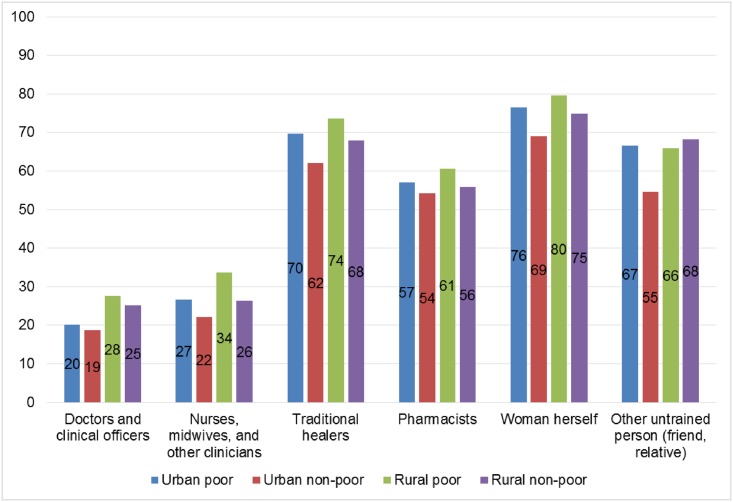
Proportion experiencing complications by provider type, Knowledgeable Informant Survey, Malawi 2015.

Knowledgeable informants estimated that urban non-poor women obtain abortions from doctors, clinical officers, nurses, midwives, or other clinicians more commonly than poor women or rural women, and that urban non-poor women self-induce abortion less commonly than other women (Fig 1). In addition, KIS respondents estimated that poor women and rural women see traditional healers more commonly than urban non-poor women (Fig 1). These experts estimated fewer complications among women procuring abortions from doctors, clinical officers, nurses, midwives and other clinicians (ranging from 19% to 34%, regardless of urban/rural or poor/non-poor status), versus those procuring abortions from traditional healers, pharmacists, self-induction, or from an untrained individual (ranging from 54% to 80%, regardless of urban rural or poor/non-poor status) (Fig 2). We estimated that 38% of women who had induced abortions in Malawi in 2015 were treated at a health facility; corresponding to multipliers that ranged from 2.3 in the Central East zone to 3.5 in the Southwest zone (Table 4).

**Table 4:** Estimated distribution of induced abortions receiving post-abortion care, and estimated percentage of all induced abortions, Knowledgeable Informant Survey, Malawi 2015.

	Estimated # induced abortions receiving PAC (weighted), adjusted 95% CI*	Multiplier	Estimated number of induced abortions, adjusted 95% CI	% of induced abortions by zone	Abortion rate, 95% CI, by zone	Abortion rate, 95% CI, by region**	Abortion ratio, 95% CI, by zone	Abortion ratio, 95% CI, by region
**National**	**51,693 (44,429–58,959)**	2.7*	**141,044 (121,161–160,928)**	**100%**	**38 (32–43)**	**38 (32–43)**	**23 (20–26)**	**23 (20–26)**
North	12,210 (11,298–13,123)	2.5	30,897 (28,588–33,206)	21.9%	61 (57–66)	61 (57–66)	36 (34–39)	35 (32–38)
Central-West	11,745 (9,707–13,784)	2.4	28,189 (23,298–33,081)	20.0%	28 (23–32)	29 (24–35)	17 (14–20)	18 (15–21)
Central-East	8,515 (7,060–9,970)	2.3	19,496 (16,165–22,827)	13.8%	33 (27–38)		19 (16–23)	
Southwest	11,567 (9,742–13,393)	3.5	40,232 (33,883–46,581)	28.5%	46 (39–53)	39 (33–45)	29 (25–34)	24 (20–28)
Southeast	7,656 (6,622–8,690)	2.9	22,230 (19,227–25,233)	15.8%	30 (26–34)		18 (16–21)	

*The national estimated number of abortions is the sum of the regional estimates. The national multiplier was not used in this calculation and is presented for informational purposes only.

** Malawi is divided into three administrative regions: Northern, Central, and Southern. The Ministry of Health divides the country into five administrative zones: the Southern region is comprised of the Southwest and Southeast zone, the Central region is comprised of the Central-West and Central-East zone, and the Northern region is comprised of the North zone.

### Induced abortion estimate, rate, and ratio

**Fig 3:**
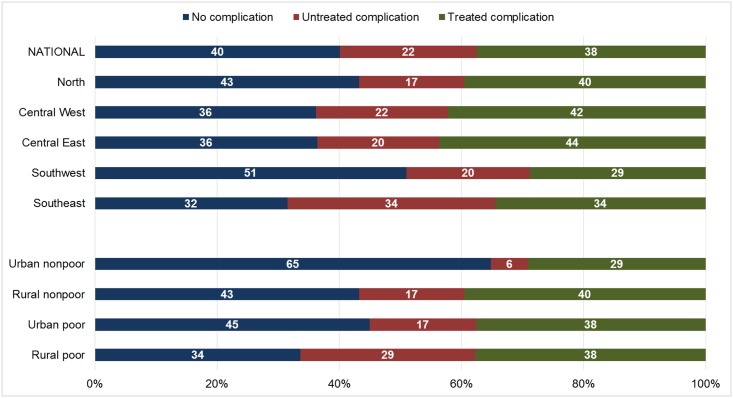
Estimated percentage distribution of abortions, by outcome, according to zone and women’s characteristics, Knowledgeable Informant Survey, Malawi 2015.

Applying zonal multipliers to the estimated number of induced abortions receiving PAC generated an estimated 141,044 induced abortions in Malawi in 2015 (95% CI: 121,161 to 160,928) (Table 4). The Southwest zone contributed the largest share of all induced abortions nationally (28.5%), and the Central-East zone contributed the smallest share (13.8%). Overall, 40% of women who had an induced abortion in Malawi in 2015 were estimated to have no complication, 22% were estimated to have untreated complications, and 38% were estimated to have a treated complication (Fig 3). The estimated proportion experiencing no complications following an induced abortion was greatest in the Southwest zone (51%) and smallest in the Southeast zone (32%), and was greatest among urban non-poor women (65%) and smallest among rural poor women (34%).

In Malawi in 2015, there were 38 induced abortions per 1,000 women aged 15–49 (95% CI: 32 to 43). The abortion rate was highest in the North zone (61, 95% CI: 57–66) and lowest in the Central-West zone (28, 95% CI: 23–32). In 2015, there were 23 induced abortions per 100 live births nationally (95% CI: 20 to 26). By zone, the abortion ratio was highest in the North (36, 95% CI: 34–39) and lowest in Central-West (17, 95% CI: 14–20).

### Unintended pregnancy

**Fig 4:**
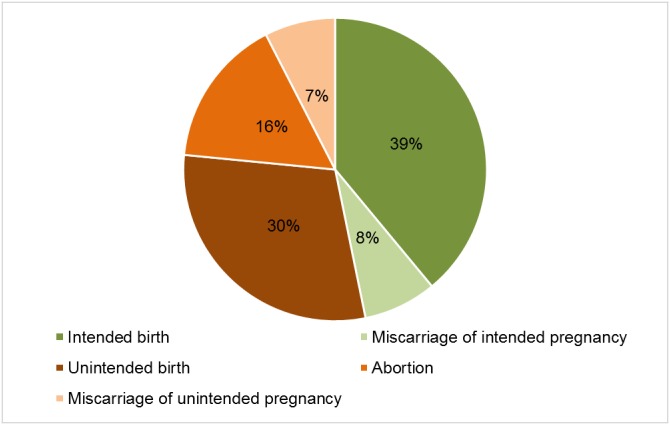
Estimated distribution of pregnancy outcomes, Malawi 2015 HFS and KIS and 2010 Malawi Demographic and Health Survey.

In Malawi in 2015, an estimated 886,161 pregnancies occurred (comprised of 609,177 births, 135,940 miscarriages, and 141,044 abortions). This translates to an overall pregnancy rate of 238 pregnancies per 1,000 women aged 15–49 (Table 5). The unintended pregnancy rate was 126 per 1,000 women aged 15–49 nationally. The North zone had the highest unintended pregnancy rate (135, 95% CI: 130–140) and Central West had the lowest (117, 95% CI: 112–123). Overall, 53% of pregnancies in Malawi were unintended, and of all unintended pregnancies, 30% ended in abortion. The percentage of unintended pregnancies ending in abortion ranged from 24% in the Central West zone to 45% in the North zone. Among all pregnancies in Malawi, an estimated 16% ended in abortion, 15% in miscarriage, 30% in unintended birth, and 39% in intended birth (Fig 4).

**Table 5:** Estimated pregnancies (overall and unintended) in Malawi, 2015.

	# pregnancies		Pregnancy rate		Unintended pregnancy rate		% of pregnancies that are unintended	
	By zone	By region*	By zone	By region	By zone	By region	By zone	By region
**National**	**886,161**	**882,056**	**238**	**238**	**126**	**126**	**53%**	**53%**
North	135,807	134,427	269	269	135	135	50%	50%
Central-West	226,109	368,612	222	228	117	123	53%	54%
Central-East	142,503		239		132		55%	
Southwest	209,815	381,742	241	237	134	127	56%	54%
Southeast	171,926		233		119		51%	

* Malawi is divided into three administrative regions: Northern, Central, and Southern. The Ministry of Health divides the country into five administrative zones: the Southern region is comprised of the Southwest and Southeast zone, the Central region is comprised of the Central-West and Central-East zone, and the Northern region is comprised of the North zone.

## Discussion

This study provides updated estimates of induced abortion in Malawi, nationally and by zone, and suggests 38 (95% CI: 32–43) induced abortions per 1,000 women aged 15–49 in 2015. This is substantially higher than the 2009 estimate (23, range 17–30) [18], but similar to other recent estimates from nearby countries, including Tanzania (36), Uganda (39), and regional estimates for Eastern and Southern Africa (34–35) [1;22;27]. However, these estimates are lower than those in Kenya (48) and higher than those in Rwanda (25) [28;29].

While our estimated abortion rate in 2015 was 38 induced abortions per 1,000 women, the 2009 study found an estimated abortion rate of 23. While the 2009 and 2015 Malawi abortion incidence studies both used a general AICM approach, they contained key methodological differences, particularly in specific sampling and calculation procedures, as detailed in the **Appendix (Table A in** S1 File**)**. Thus, we cannot quantify how much of the difference in estimates is attributable to a potential actual increase in induced abortion during the intervening 6 years, as opposed to methodological differences between the 2009 and 2015 studies. The estimated number of women treated in facilities for complications of induced abortion was 177% higher in 2015 than in 2009, though the percent share remained similarly distributed geographically (**Table D in** S1 File). The estimated number of induced abortions slightly more than doubled (104% increase), with the largest difference in the Southern region (131%). The regional distribution of the percent share of estimated induced abortions remained similar in the North (19.5% in 2009 vs. 21.9% in 2015), was lower in the Central region (41.9% vs. 33.8%), and was slightly higher in the South (38.6% vs. 44.3%) (**Table F in** S1 File). As in 2009, the estimated abortion rate and ratio was highest in the Northern region. (**Table G in** S1 File)

Limitations of the AICM have been detailed elsewhere [20]. Given the assumptions required for calculation, results should be viewed as approximate estimates, rather than exact measures. For example, one assumption relates to the validity of constructing a multiplier based on informed opinions from key informants. In this study, we derived information from a large sample (considering the small size of the country) of carefully selected informants, and ensured inclusion of individuals familiar with abortion in rural areas. Another assumption is that late miscarriages equal 3.41% of births [24]. If this figure is higher in Malawi, our current estimates for PAC cases due to late miscarriage would be underestimated, and our current estimates for induced abortions and the abortion rate would be overestimated. For example, if we double our assumptions about this percentage (6.82%), the abortion rate would fall to 26. Lacking data on the proportion of women who follow up on referrals, we used an estimate of 75% based on data from Tanzania. If we assume a higher follow up rate such as 95%, the overall abortion rate would drop slightly to 36. We also assume that women experiencing miscarriage in the first trimester would not be treated for PAC, although it is possible that this may occur in a small number of cases, due to infection or other reasons. An additional assumption is that the distribution by wealth and residential status (urban vs. rural) of women having an induced abortion is similar to the distribution of all women in Malawi, although it is possible that differences in rates of unintended pregnancy, or propensity to abort, may vary by wealth and residence. An additional limitation of this approach is that it does not provide information on the characteristics of individual women seeking PAC or the severity of abortion complications, though studies in prior years have investigated these issues with additional data collection efforts [16;30].

In this study, response rates were high among central, district, and rural/community hospitals, as well as maternity units and health centers (between 93–100%), but somewhat lower among clinics (62%) and “other” hospitals (70%). However, of all sampled facilities, 7 health centers, 11 clinics, and 4 “other” hospitals refused to provide an interview because they did not provide PAC. Had these facilities agreed to be interviewed, our results would have remained unchanged, but our response rate would have been 95% overall (including 97% among health centers, 92% among clinics and 81% among “other” hospitals). Thus, representativeness for most facility types was high. We identified 4 clinics that closed prior to our fieldwork; if those facilities provided PAC during a portion of 2015, our PAC totals (and thus our abortion rate) would be underestimates. A key limitation is the lack of clarity around the extent to which misoprostol is used to induce abortion in Malawi. While 41% of KIS respondents believed misoprostol might be being used to induce abortion, they indicated that any such use would be largely confined to women in urban areas, who represent less than 20% of the female population of Malawi. Increases in use of misoprostol for induced abortion might decrease the rate of actual complications, or could artificially increase estimated PAC caseloads (if women using medication abortion visited a facility for normal bleeding and was counted as a PAC case), and thus impact overall abortion estimates in opposing directions. Future studies would benefit from development of methods to more accurately determine the extent and impact of misoprostol use on AICM estimates.

The percent of married women currently using a modern contraceptive method in Malawi increased from 28% in 2004 [31], to 42% in 2010 [26], to 58% in 2015 [19]. Among sexually active unmarried women, these estimates increased from 24% in 2004 [31] to 46% in 2010 [26], but fell slightly to 43% in 2015 [19]. Since increased contraceptive use may be expected to reduce unintended pregnancy, simultaneous increases in contraceptive use and abortion rates during the same time period may seem paradoxical at first glance. However, recent increases in contraceptive use in Malawi have not translated into lower fertility rates [26]. Several plausible explanations for this seeming disconnect have been put forward, including that contraceptive increases may be occurring among women at lower risk of pregnancy, or potentially due to contraceptive discontinuation [32]. Furthermore, simultaneous increases in contraception and abortion can occur in the context of concurrent increases in desire for smaller family size [33]. In Malawi, ideal family size among all women decreased substantially between 2000 and 2004 (from 5 to 4.1), but remained similar between 2004 and 2010 [26], and estimates for ideal family size from the 2015 DHS are not yet available [19]. Thus, recent increases in contraceptive use in Malawi may not be incompatible with potential simultaneous increases in abortion. Given that most abortions result from unintended pregnancies, policies and programs should continue to promote contraceptive use and help couples use their methods correctly and consistently.

Since 2003, the Ministry of Health has made efforts to increase the number of public facilities providing PAC, including a focus on facility upgrades and provider training [30]. Although use of manual vacuum aspiration (MVA) for treatment of incomplete abortion is recommended by WHO and favorable to use of dilation and curettage (D&C) [34], recent studies have documented declining MVA use in PAC in selected hospitals in Malawi [35]. In our study, when asked how treatment for abortion complications could be improved, 74% of HFS respondents said “have more commodities available” while 64% said “have more trained people available” (data not shown), which supports findings from a qualitative study of PAC providers to understand declines in use of MVA in recent years [36]. Addressing availability of equipment, providing regular training, and addressing staff shortages could improve PAC in Malawi [36].

The findings of our study should be carefully considered in Malawi’s ongoing effort to reduce maternal morbidity and mortality and improve public health using evidence-based, cost-effective approaches. A costing study noted that treating 18,600 women for complications of induced abortion in public health facilities in 2009 represented a significant burden to the health system, and estimated that a liberalized abortion law and access to safe abortion in public health facilities would yield a 20–30% decrease in PAC costs [37]. We estimate that more than 140,000 Malawian women risked their health and lives in 2015 by seeking a clandestine abortion under the current restrictive abortion law. Allowing for additional indications for safe and legal induced abortion could reduce the number of women who experience complications from unsafe abortion. In turn, this could save women’s lives, protect their well-being, conserve scarce financial resources [37], and prevent intergenerational impacts of maternal mortality on children, extended families, and communities throughout Malawi [38].

## Supporting information

S1 File Appendix text and tables. (DOCX)

S2 File Questionnaire: Health Facilities Survey. (PDF)

S3 File Questionnaire: Knowledgeable Informant Survey on the condition of abortion in Malawi. (PDF)
