# Supplementary material for: Incidence of induced abortion in Malawi, 2015
Source: PLoS One. 2017 Apr 3;12(4):e0173639. doi: 10.1371/journal.pone.0173639 (PMC5378324; doi:10.1371/journal.pone.0173639)
Supplement: S1 File — (DOCX) [file pone.0173639.s001.docx]

**APPENDIX**

**Text A**

*Calculations to obtain the zonal multipliers can be expressed as:*

A_jk_ = % of women who have an abortion complication, by subgroup and zone

B_jk_ = % of women with complications who obtain care in a health facility, by subgroup and zone

C_jk_ = % obtaining treatment for complications in a health facility, among all women having abortions, by subgroup and zone

*D_k_ =* weighted % of women who had an abortion who received treatment

W_jk_ =distribution of the population of women, by subgroup and zone

M_k_ = zonal multiplier

Where: j=subgroup (rural poor, rural nonpoor, urban poor, urban nonpoor) and k=zone (North, Central-West, Central-East, Southwest, Southeast)

Then:

C_jk_=(A_jk_*B_jk_)

*D_k_=Sum(W_jk_ * C_jk_)*

M_k_=1/D_k_

*To obtain the estimated number of induced abortions, the multiplier is applied to the estimated number of induced abortions receiving PAC:*

E_k_=Estimated annual PAC caseload, weighted by the inverse of the product of the sampling fraction and the response rate for each facility type, by zone

F_k_=Estimated number of double-counted referrals, weighted, by zone

G_k_=Estimated number of late miscarriages treated for PAC, by zone

H_k_ = (E_k_ - (F_k_+G_k_)) (i.e., estimated number of induced abortions receiving PAC, weighted by zone)

I_k_ = H_k_*M_k_ (i.e., estimated number of induced abortions by zone)

J=Sum(I_k_) (i.e., total number of induced abortions in the country)

**Table A. Key methodological differences between 2009 and 2015 Malawi AICM studies.**

|  | **2009 [16]** | **2015 (current study)** | **Implication** |
| --- | --- | --- | --- |
| HFS, sampling procedures | Began with list of health facilities in Malawi (n=893) taken from November 2008 edition of Malawi Government Gazette, supplemented by lists of facilities associated with BLM and MoHs reproductive health unit. Facilities that clearly did not provide maternal health care (i.e., Malawi Defense Force health facilities and specialized clinics (i.e., mental health clinics)) were excluded. Lists of remaining facilities were given to Ministry of Health zonal health supervisors, who indicated that 427 facilities on the list were likely to be providing PAC. Other facilities were excluded. This resulted in a sampling frame of 427 facilities (**Table B**). | Began with list of health facilities from 2013-2014 Malawi Service Provision Assessment; designed to be a census of all health facilities throughout the country (n=977 facilities, representing 92% of all known health facilities). 50 facilities that clearly could not provide PAC services (dental offices, prison clinics, podiatry clinics, HIV clinics, orthopedic clinics, etc.) were excluded, along with 46 dispensaries and 20 health posts (since PAC services are not offered at this level of health care facility in Malawi). This resulted in sampling frame of 861 facilities (**Table B**). | If PAC-providing facilities were believed by zonal supervisors not to be providing PAC and therefore excluded, PAC caseloads (and thus abortion rate), may have been underestimated in 2009. In 2015, we included all randomly selected facilities in our sample, and directly interviewed them to determine if they provide PAC. |
| HFS, facility types eligible for inclusion | Included all public hospitals, public health centers, NGO hospitals, NGO health centers, and private hospitals “thought to be providing PAC”, as well as 33.3% of private clinics (resulted in target list of 269 facilities; of which 162 were providing PAC and participated in the HFS). (**Table B**). | Included all hospitals (central, district, rural/community, other) and maternity units, 40% of health centres, and 12% of clinics – including public, private, or NGO owned facilities at all levels. (resulted in target list of 334 facilities; of which 294 participated [202 of those provided PAC]). (**Table B**). | Unlike in 2015, the 2009 HFS did not include private health centers, public or NGO clinics, or maternity units, which may have impacted estimation of national PAC caseloads in 2009 (**Table C**). |
| Triangulation of HFS data | In addition to the HFS survey, incorporated information from a prospective survey that collected information on PAC caseloads in facilities over the course of 30 days, as well as logbook data, to triangulate information on PAC caseloads from the HFS. | Did not incorporate a prospective survey or logbook data to triangulate estimates on PAC caseloads from HFS data. | Lack of prospective data collection in 2015 is unlikely to explain differences in abortion rates between 2009 and 2015: recalculating 2009 estimates without information from prospective component, increased the overall abortion rate from 23 to 25, well below our 2015 estimate of 37. (**Table C**) |
| Denominator of women 15-44 vs. 15-49 | Denominator included women ages 15-44, excluding the 45-49 age group. Had the denominator instead been women ages 15-49, the abortion rate (using only HFS data on PAC cases) would have been 23. | Denominator included women ages 15-49. Had the denominator included only women ages 15-44, the abortion rate would have been 40. To summarize, when the denominator consists of women ages 15-49, the 2009 rate is 23 and the 2015 rate is 37. When the denominator includes only women ages 15-44, the 2009 rate is 25 and the 2015 rate is 40. | We think it is appropriate to include the group of women 44-49 in the denominator, since they have a non-negligible birth rate of 26 per 1000 in Malawi, so may also contribute to a proportion of abortions. |
| HFS, calculation of late miscarriages | Assumed that number of late miscarriage equals 3.41% of live births. Assumed that the proportion of women who given birth at a health facility in a recent year is equivalent to the proportion of women who will obtain care for a late miscarriage (54% nationally, 51-61% across regions) (**Table D**). | Assumed that number of late miscarriage equals 3.41% of live births. Used information from KIS survey (separately for urban and rural women), to estimate the proportion of late miscarriages treated in a health facility (median: 90% among urban women, 70% among rural women) (**Table D**). | Differences in assumptions about the proportion of women who obtain care for a late miscarriage may impact the number of estimated miscarriages subtracted from total PAC caseloads, thereby impacting estimates of induced abortions, abortion rate, and abortion ratio. |
| HFS, subtraction of referrals | Did not subtract potentially double-counted referral cases (**Table D**). | Subtracted potentially double-counted referral cases (**Table D**). | PAC caseloads (and thus induced abortion), may have been over-estimated in 2009 if some cases were double-counted at more than one facility (**Table D**). |
| KIS, sample size, response rates, and knowledge of rural areas | Produced a list of 123 potential respondents, of whom 56 were successfully interviewed. Most (61%) worked in one of Malawi’s two major cities (Lilongwe or Blantyre). | Produced a list of 125 key informants, who represented 27 districts in Malawi and had more substantial knowledge about the situation of abortion in rural areas (where the 82% of the population of Malawi lives). While 16% of our respondents worked in urban areas, 42% worked in rural areas and another 42% worked in both urban and rural areas. Interviewers determined that the respondent knew about abortion in rural areas very well or moderately well in 91% of interviews. | Calculation of multipliers in 2015 is based on a larger sample of respondents, and a respondent pool with more familiarity with the situation of abortion for women living in rural areas of Malawi. (**Table E**) |
| KIS, Calculation of zonal multipliers | A national multiplier was calculated (3.6) and applied to zones. | Zonal multipliers were calculated (ranging from 2.3-3.5) and applied to specific zones. | Calculations in 2015 were more specific by geographic zone (**Tables E, F, G**). |

**Table B. Differences in sampling between 2009 and 2015 Malawi AICM studies.**

| **2009** | | | | | | | |
| --- | --- | --- | --- | --- | --- | --- | --- |
| Facility Type | # facilities | # facilities **thought by MOH to provide** PAC | % of target facilities sampled | # sampled facilities | # sampled facilities providing PAC | # facilities that participated in HFS | % of facilities participating |
| Public hospital | 56 | 50 | 100% | 50 | 47 | 47 | 100 |
| Private hospital | 2 | 2 | 100% | 2 | 2 | 2 | 100 |
| NGO hospital | 41 | 33 | 100% | 33 | 30 | 29 | 97 |
| Public health center | 405 | 64 | 100% | 64 | 46 | 45 | 98 |
| NGO health center | 152 | 41 | 100% | 41 | 35 | 34 | 97 |
| Private clinic | 237 | 237 | 33.3% | 79 | 6 | 5 | 83 |
| All | 893 | 427 |  | 269 | 166 | 162 | 98 |
| **2015** | | | | | | | |
| Facility Type | # facilities | # facilities **possibly providing** PAC | % of facilities sampled | # facilities sampled | # facilities that participated in HFS | % of facilities participating in HFS | # sampled facilities providing PAC |
| Central hospital | 4 | 4 | 100% | 4 | 4 | 100% | 4 |
| District hospital | 24 | 24 | 100% | 24 | 24 | 100% | 23 |
| Rural/community hospital | 41 | 41 | 100% | 41 | 39 | 95% | 39 |
| Other hospital | 47 | 40 | 100% | 40 | 28 | 70% | 22 |
| Health centre | 473 | 467 | 40% | 187 | 174 | 93% | 103 |
| Clinic | 317 | 281 | 12% | 34 | 21 | 62% | 10 |
| Maternity unit | 4 | 4 | 100% | 4 | 4 | 100% | 1 |
| Dispensary | 47 | 0 | 0% | 0 | 0 | -- | -- |
| Health post | 20 | 0 | 0% | 0 | 0 | -- | -- |
| All | 977 | 861 |  | 334 | 294 | 88% | 202 |

**Table C. Differences in estimated number of post-abortion care cases, Malawi 2009 and 2015**

| **Estimated # PAC cases, 2009 and 2015** | | | | | |
| --- | --- | --- | --- | --- | --- |
| **2009** | | **2015** | | | |
| Region | N, 95% CI* | Zone | N, 95% CI | Region | N, 95% CI |
| North | 5,258 | North | 15,473 (14,561 - 16,385) | North | 15,473 (14,561 - 16,385) |
| Central | 12,138 | Central-West | 18,360 **(**16,322 - 20,398**)** | Central | 30,792 (27,299 - 34,285) |
|  |  | Central-East | 12,432 **(**10,977 - 13,887**)** |  |  |
| South | 12,101 | Southwest | 16,291 (14,466 - 18,117) | South | 28,062 (25,203 – 30,922) |
|  |  | Southeast | 11,771 (10,737 - 12,805) |  |  |
| **National** | **29,497 (25,635-33,359)** | **National** | **74,328 (67,063 - 81,593)** | **National** | **74,328 (67,063 - 81,593)** |

* 95% CI not provided by region in 2009 study.

**Table D. Differences in estimated number of post-abortion care cases stemming from induced abortion, Malawi 2009 and 2015**

| **Estimated # PAC cases stemming from induced abortion, 2009 and 2015** | | | | | | | | |
| --- | --- | --- | --- | --- | --- | --- | --- | --- |
| **2009**  **(after subtracting late miscarriages)** | | | **2015**  **(after subtracting late miscarriages and referrals)** | | | | | **% increase, 2009 to 2015** |
|  | By region | |  | By zone | | By region | |  |
|  | N | % |  | N | % | N | % |  |
| North | 3,640 | 19.5% | North | 12,210 | 23.6 | 12,210 | 23.6 | 235% |
| Central | 7,828 | 41.9% | Central-West | 11,745 | 22.7 | 20,260 | 39.2 | 159% |
|  |  |  | Central-East | 8,515 | 16.5 |  |  |  |
| South | 7,218 | 38.6% | Southwest | 11,567 | 22.4 | 19,223 | 37.2 | 166% |
|  |  |  | Southeast | 7,656 | 14.8 |  |  |  |
| **National** | **18,686** | **100%** | **National** | **51,693** | **100%** | **51,693** | **100%** | **177%** |

**Table E. Multipliers calculated from survey of key informants, Malawi 2009 and 2015**

| **AICM multipliers in 2009 and 2015** | | |
| --- | --- | --- |
|  | **2015** | **2009** |
| North | 2.5 | 3.6 (2.6-4.6) |
| Central-West | 2.4 | 3.6 (2.6-4.6) |
| Central-East | 2.3 | 3.6 (2.6-4.6) |
| Southwest | 3.5 | 3.6 (2.6-4.6) |
| Southeast | 2.9 | 3.6 (2.6-4.6) |
| **National** | **2.7*** | 3.6 (2.6-4.6) |

*National multiplier not used in 2015 calculations; presented for informational purposes only.

**Table F. Estimated number of induced abortions in Malawi, 2009 and 2015**

| **2009** | | | **2015** | | | | **2009 to 2015** |
| --- | --- | --- | --- | --- | --- | --- | --- |
| **Region** | **Estimated number of induced abortions (range)** | **% of induced abortions by region** | **Zone** | **Estimated number of induced abortions (adjusted 95% CI)** | **% of induced abortions by zone** | **% of induced**  **abortions by**  **region** | **% increase** |
| North | 13,104  (9,464-16,744) | 19.5% | North | 30,897  (28,588 - 33,206) | 21.9% | 21.9% | 136% |
| Central | 28,181  (20,353-36,009) | 41.9% | Central-West | 28,189  (23,298 - 33,081) | 20.0% | 33.8% | 69% |
|  |  |  | Central-East | 19,496  (16,165 - 22,827) | 13.8% |  |  |
| South | 25,985  (18,767-33,203) | 38.6% | Southwest | 40,232  (33,883 - 46,581) | 28.5% | 44.3% | 140% |
|  |  |  | Southeast | 22,230  (19,227 - 25,233) | 15.8% |  |  |
| **National** | **67,270**  **(48,584-85,956)** | **100%** | **National** | **141,044**  **(121,161 - 160,928)** | **100%** | **100%** | 110% |

**Table G. Estimated abortion rate and ratio, Malawi, 2009 and 2015**

|  | **Abortion rate** | | **Abortion ratio** | |
| --- | --- | --- | --- | --- |
|  | **2009, by region**  **(range)** | **2015, by zone**  **(95% CI)** | **2009, by region**  **(range)** | **2015, by zone**  **(95% CI)** |
| North | 34.8 (25.1-44.4) | 61 (57-66) | 16.7 (12.1-21.4) | 36 (34-39) |
| Central-West | 23.3 (16.8-29.8) | 28 (23-32) | 11.3 (8.2-14.5) | 17 (14-20) |
| Central-East |  | 33 (27-38) |  | 19 (16-23) |
| Southwest | 20.0 (14.5-26.5) | 46 (39-53) | 10.1 (7.3-12.9) | 29 (25-34) |
| Southeast |  | 30 (26-34) |  | 18 (16-21) |
| **National** | **23.3 (16.8-29.8)** | **38 (32-43)** | 11.5 (8.3-14.7) | **23 (20-26)** |
