# Supplementary material for: Incidence of induced abortion in Malawi, 2015
Source: PLoS One. 2017 Apr 3;12(4):e0173639. doi: 10.1371/journal.pone.0173639 (PMC5378324; doi:10.1371/journal.pone.0173639)
Supplement: S2 File — (PDF) [file pone.0173639.s002.pdf]

UNIVERSITY OF MALAWI

STUDY OF ABORTION AND WOMEN'S HEALTH IN MALAWI

HEALTH FACILITIES SURVEY

GUTTMACHER INSTITUTE

IDENTIFICATION

NAME OF HEALTH FACILITY: ______________________ **ID number** [| | | |]

M1 HEALTH ZONE A. North B. Central-West C. Central-East D. Southwest E. Southeast

M2 DISTRICT .... [ ]

M3 **TYPE OF HEALTH FACILITY (Please circle the appropriate number.)**

1	CENTRAL HOSPITAL	4	OTHER HOSPITAL
2	DISTRICT HOSPITAL	5	HEALTH CENTRE
3	RURAL/COMMUNITY HOSPITAL	6	CLINIC
		7	MATERNITY

M4 **OWNERSHIP (Ask respondent, and circle the appropriate number.)**

1	Public/government
2	Private for-profit
3	Private not-for-profit
4	CHAM
5	BLM
6	Other NGO: ______________________
7	Other: ______________________

M5 INTERVIEWER'S NAME ______________________ [ ]

M6 SUPERVISOR'S NAME ______________________ [ ]

M7 DATE OF INTERVIEW [|] [|] [2|0|1|5]
day month Year

M8 TIME STARTED [|]h [|]m

Complete rest of this page after the informed consent (and interview, if accepted) is complete.

M9 TIME ENDED [|]h [|]m

M10 INTERVIEW OUTCOME

1	Completed
2	Refused - no incentive
3	Refused - any other reason
4	Incomplete
5	Not available for interview
6	Health facility not functional (closed, nonexistent)

Commentary: ______________________

____

INTERVIEWER SIGNATURE (after interview): ______________________

QUALITY CONTROL SIGNATURE (after QC check): ______________________

DATA MANAGER SIGNATURE (after data entry): ______________________

Module 1: Basic Information

No.	Questions and Filters				Responses
101	What is your position at this facility?<br><br>**[Interviewer: circle the category that applies to the respondent.]**			01	Gynecologist-obstetrician
				02	General practitioner
				03	Clinical officer
				04	Medical assistant
				05	Nurse-midwife technician/enrolled nurse midwife
				06	Registered nurse midwife
				07	Other nurse (e.g,. community health nurse)
				96	Other (specify): ________
102	*Gender of the respondent*<br>**[Interviewer: circle the category that applies to the respondent. Do not ask this question aloud.]**			1	Male
				2	Female
103	How old are you?				Years
104	How many years have you worked in your current profession?<br><br>**[If less than 12 months, enter "0".]**				Years
105	How many years have you worked in this facility?<br><br>**[If less than 12 months, enter "0".]**				Years
106	Which of the following gynecological and obstetric services does this facility offer?<br><br>**[Interviewer: Please read out all relevant categories. Multiple responses are allowed.]**		**Gynaecological obstetric services offered:**		
			**No**	**Yes**	
		a	0	1	Gynaecological services
		b	0	1	Maternity and delivery services
		c	0	1	Antenatal care
		d	0	1	Postnatal care
		e	0	1	Family planning services
		f	0	1	HIV testing and counseling
		g	0	1	ART services
		h	m	1	STI services
		i	0	1	PMTCT services
		j	0	1	Cervical cancer screening
		m	0	1	Immunization
		l	0	1	Other (specify, limit to 2-3): ________ ________
		m	0	1	None of the above

			No	Yes	
107	Which of the following items are available and functional at this facility?<br><br>**[Interviewer: Please read out all relevant categories. Multiple responses are allowed.**<br><br>**NOTE: Functionality applies to day of interview, that is, if the equipment is not working that day, it is not functional (0). For electricity, if there is a temporary power outage but the electricity will come back on when ESCOM returns it, then electricity is functional; otherwise, it is not.]**	a	0	1	Operating room
		b	0	1	Instrument preparation room
		c	0	1	Outpatient department (OPD)
		d	0	1	Recovery room
		e	0	1	Drug store
		f	0	1	Laboratory
		g	0	1	Blood transfusion service
		h	0	1	Intensive care unit (ICU)
		i	0	1	Sonogram (scanning)
		j	0	1	Local anaesthetics (numbs one area)
		k	0	1	General anaesthetics (sedates patient)
		l	0	1	Water
		m	0	1	Electricity / solar power
		n	0	1	Blood pressure machine
		o	0	1	Intravenous fluids
		p	0	1	Telephone or radio communication
108	Which of the following essential drugs and tests are available at this facility?<br><br>**[Interviewer: Read each item on the list.**<br><br>**NOTE: Again, availability refers to the day of interview. If the drug/test is not available the day you are there, please code it as No (0).]**	a	0	1	Oxytocin/ergometrine
		b	0	1	Antibiotics
		c	0	1	Analgesics
		d	0	1	Sedatives
		e	0	1	IV fluids
		f	0	1	Hemoglobin test
		g	0	1	Syphilis test
		h	0	1	HIV test
		i	0	1	Pregnancy test
		j	0	1	Blood grouping test

Module 2: Postabortion Care

Now, I would like to ask you some questions regarding ***medical care for patients treated at this facility with abortion complications, irrespective of whether the abortion was spontaneous or induced.*** Among other things, I will ask about ***the number of patients treated for such complications at your facility.*** By abortion complications, we are referring to those complications that are severe enough to **need treatment** in a health facility. Abortion complications, as defined here, include not only the **extremely serious cases** such as those with sepsis or a perforated uterus, but also those cases which are termed "incomplete abortions," which are usually identified by heavy bleeding, and which present a somewhat less severe health risk to the woman, but which, nevertheless, need hospitalization. In answering the following set of questions concerning abortion complications, please keep this definition in mind.

[Interviewer: Please note that the abortion-complication questions relate to both spontaneous and induced abortions. You should reiterate this as often as possible while completing this section.]

The following questions refer to all cases of abortions that are underway (spontaneous or induced), incomplete abortions and postabortion care.

201	On average about how many deliveries take place at your facility each month?<br><br>**[Interviewer: if respondent can't answer for average month, probe for the total number in 2014.]**	a. Deliveries per month<br>**OR**<br>b. Total number of deliveries during the 2014 calendar year			
202	Does this facility treat women with spontaneous abortions or complications from induced abortions?			0	No -> **[END INTERVIEW.]**
				1	Yes
203	In which areas of this health facility are patients with abortion complications treated?<br><br>**[Interviewer: Please read out the list of wards and circle all that apply.]**		No	Yes	
		a.	0	1	General outpatient clinic
		b.	0	1	Outpatient maternal-child clinic
		c.	0	1	Outpatient gynaecological clinic
		d.	0	1	General surgical ward
		e.	0	1	Gynaecological/Obstetric ward
		f.	0	1	Intensive care unit (ICU)
		g.	0	1	Emergency ward
		h.	0	1	Other ward (specify):
204	In your facility, are post-abortion patients treated as outpatients (i.e., are not admitted, day cases) only, inpatients only (i.e., are admitted, stay overnight), or both?			1	Inpatient only **-> GO TO Q207**
				2	Outpatient only
				3	Both

205	During **an average month,** about how many post-abortion care patients would you estimate are treated as **outpatients** in this facility as a whole? Please remember to include all patients treated for abortion complications, whether they are spontaneous or induced.<br><br>**[Interviewer : Please probe to elicit a response for an average month; if respondent is <u>not</u> able to provide you with that estimate, then probe for the number of outpatients on average per year. Specify that this is a full calendar year (i.e. from January to December). Please reiterate to the respondent that the number is for spontaneous and induced abortion patients, and should take into consideration all wards of the facility.]**	a. Number of postabortion care outpatients in an average month [ ][ ][ ][ ]<br><br>**OR**<br><br>b. Number of postabortion care outpatients in an average year [ ][ ][ ][ ]
206	In **the past month**, about how many post-abortion care patients were treated as **outpatients** in this facility as a whole? Please remember to include all patients treated for abortion complications, whether they are due to spontaneous or induced abortions.<br><br>**[Interviewer : Please probe to elicit a response for the past month; if respondent is not able to provide you with that estimate, then probe for the number of outpatients during the year 2014. Please reiterate to the respondent that the number should take into consideration all wards of the facility.]**	a. Number of postabortion care outpatients in the past month [ ][ ][ ][ ]<br><br>**OR**<br><br>b. Number of postabortion care outpatients last calendar year (2014) [ ][ ][ ][ ]

**[Interviewer: See Q. 204. If the Health Facility provides ONLY outpatient services, go to Q. 209.]**		
207	During **an average month,** about how many post-abortion care patients would you estimate were treated as **inpatients** at this facility as a whole? Please remember to include all patients treated for abortion complications, whether they are due to spontaneous or induced abortions.<br><br>**[Interviewer : Please probe to elicit a response for an average month; if respondent is not able to provide you with that estimate, then probe for the number of inpatients on average per year. Specify that this is a full calendar year (i.e. from January to December). Please reiterate to the respondent that the number is for spontaneous and induced abortion patients, and should take into consideration all wards of the facility.]**	a. Number of postabortion care inpatients in an average month ☐☐☐☐<br><br>**OR**<br><br>b. Number of postabortion care inpatients in an average year ☐☐☐☐
208	In t**he past month**, about how many post-abortion care patients were treated as **inpatients** in this facility as a whole? Please remember to include all patients treated for abortion complications, whether they are due to spontaneous or induced abortions.<br><br>**[Interviewer : Please probe to elicit a response for the past month; if respondent is not able to provide you with that estimate, then probe for the number of inpatients during the year 2014. Please reiterate to the respondent that the number should take into consideration all wards of the facility.]**	a. Number of postabortion care inpatients in the past month ☐☐☐☐<br><br>**OR**<br><br>b. Number of postabortion care inpatients last calendar year (2014) ☐☐☐☐

209	**[Interviewer : Refer to the previous figures before asking the respondent about the patient totals.]**<br><br>Just to confirm what you have just told me, in an **average month** (or year), your facility treated<br><br>___ outpatients (Q205)<br>___ inpatients (Q207).<br><br>**[Interviewer: Please read out the total number of spontaneous and induced abortion patients seen at this facility in an average month (Q 205 and Q 207).]**<br><br>Is this number correct ?<br><br>**[Interviewer: If correct, please insert again at right; if not, then correct Q 205 and Q 207 and insert at right.]**	Summary **of average** per month:<br><br>a. Outpatients (Q205) ☐☐☐☐<br><br>b. Inpatients (Q207) ☐☐☐☐<br><br>**OR**<br><br>Summary **on average** per year:<br><br>c. Outpatients (Q205) ☐☐☐☐<br><br>d. Inpatients (Q207) ☐☐☐☐
210	**[Interviewer : Refer to the previous figures before asking the respondent about the patient totals.]**<br><br>Just to confirm what you have just told me, in the **past month** (or **during the year 2014),** your facility treated<br><br>___ outpatients (Q206) and<br>___ inpatients (Q208)<br><br>**[Interviewer: Please read out the total number of spontaneous and induced abortion patients seen at this facility last month (Q 206 and Q 208).]**<br><br>Is this number correct ?<br><br>**[Interviewer: If correct, please insert again at right; if not, then correct Q 206 and Q 208 and insert at right.]**	Summary of number **in past month**:<br><br>a. Outpatients (Q206) ☐☐☐☐<br><br>b. Inpatients Q208) ☐☐☐☐<br><br>**OR**<br><br>Summary of number **in year 2014**:<br><br>c. Outpatients (Q206) ☐☐☐☐<br><br>d. Inpatients (Q208) ☐☐☐☐

211	Thinking about patients you have treated at your facility in the past 1 year (12 months), what percentage of the post-abortion patients at this facility had the following complications?<br><br>**[Interviewer: Read each complication aloud. The percentages must add up to at least 100%. They may add up to more than 100% since a patient may suffer from more than one complication.]**				**%**
			A	Hemmorhagic shock	
			B	Septic shock	
			C	Sepsis	
			D	Uterine perforation	
			E	Hemorrhage	
			F	Incomplete abortion	
			G	Cervical/vaginal lacerations	
			H	Visceral injury (ie. bladder, intestine)	
			X	Other (specify): ____ ____	
					**100+**
212	a. Last year (2014), did any woman die in your facility from any of these abortion-related complications?<br><br>b. If yes, please estimate how many died?	0	No		
		1	Yes		
			[ ][ ][ ] number who died		
213	Out of all the patients you treated in the **past month**, how many were referred to your facility *after having been treated* at a lower level facility?<br><br>(ONLY if they don't know/can't estimate for past month:) Or in the year 2014?<br><br>**[NOTE: If 0, circle "Y" in Q214, then skip to Q215.]**	Total number of postabortion care patients received from another health facility in the **past month** [ ][ ][ ]<br><br>**OR**<br><br>Total number of postabortion care patients received from another health facility **in the year 2014** [ ][ ][ ]			
214	What reasons were given by the escorts or in the referral records of patients referred to you for postabortion care?<br><br>**[Interviewer: Do not prompt. Circle all that apply.]**	A	Medical reasons (fever, lower abdominal pain, hemorrhage (or anemia), perforation of uterus)		
		B	Comprehensive care		
		C	Need for blood transfusion		
		D	Lack of equipment, drugs, consumables, etc		
		E	Lack of qualified personnel/medical expertise		
		F	Family planning		
		X	Other (specify): ____		
		Y	Received no referrals		

215	Out of all the post-abortion care patients treated at your facility in the **past month**, how many have you referred to another level facility for adequate coverage, **after** having treated them for postabortion care (either as inpatients or outpatients)?<br><br>**(ONLY if they don't know/can't estimate for past month, or if they say there were no referrals in the past month:)** Or in the year 2014?<br><br>**Note:** If 0, circle "Y" in 216, then skip to Q217.	a. Total number of patients referred for postabortion care in the past month ☐☐☐<br><br>**OR**<br><br>b. Total number of patients referred for postabortion care in the year 2014 ☐☐☐	
216	What are the medical reasons (indications) for transfering a postabortion care case to a higher level facility?<br><br>**[Interviewer: Do not prompt. Circle all that apply.]**	A	Medical reasons (fever, lower abdominal pain, hemorrhage (or anemia), perforation of uterus)
		B	Comprehensive care
		C	Need for blood transfusion
		D	Lack of equipment, drugs, consumables, etc
		E	Lack of qualified personnel/medical expertise
		F	Family planning
		X	Other (specify):
		Y	Does not apply (don't refer patients)
217	What are the various procedures and measures used to treat abortion-complication patients at this facility?<br><br>**[Interviewer: Do not prompt. Circle all that apply.**	A	Evacuation with a sharp curette (D&E, D&C)
		B	MVA (manual vacuum aspiration)
		C	Electric vacuum aspiration
		D	Surgery (e.g. laparotomy, repair injured viscera, hysterectomy)
		E	Misoprostol
		F	Blood transfusion
		G	Antibiotics
		H	Injectable uterotonic (e.g. Oxytocin, ergometrine)
		I	IV fluids
		X	Other (specify):

218	In this facility, what percentage of postabortion care patients are treated by the following procedures?<br><br>**[Interviewer: If the respondent has trouble with 100%, you can ask "How many out of 10" instead, as long as you convert to percentages by multiplying each response by 10.]**<br><br>**[Interviewer: Read each procedure to the respondent. After you have completed the list, make sure all numbers add up to at least 100. There can be 0% for certain procedures, but there must be a number in each box. It is OK if the sum is more than 100 because some patients might receive more than 1 procedure.]**			**%**
		A	Evacuation with a sharp curette (D&E, D&C)	
		B	MVA (manual vacuum aspiration)	
		C	Electric vacuum aspiration	
		D	Surgery only (e.g. laparatomy)	
		E	Misoprostol	
		X	Other (specify):	
				**100+**
219	Out of every 10 women treated in this facility for abortion complications, how many, in your opinion, had a spontaneous abortion or miscarriage?		Number of spontaneous abortions out of every 10 PAC (postabortion care) cases	___ ___
220	Out of every 10 women treated in this facility for abortion complications, how many, according to your best estimate, were in the first trimester of pregnancy?		Number of 1st trimester pregnancy losses out of every 10 PAC (postabortion care)	___ ___
221	Does this facility currently possess functional manual vacuum aspiration (MVA) kits?	0	No	
		1	Yes	

Module 3: General

301	Do you consider treatment of abortion complications to be a burden for your facility?	0	No **[Go to 302]**
		1	Yes
	**If yes, ask: "In what respect does treatment of abortion complications pose a burden for your facility?"** **[Do not read. Circle all that apply.]**	A	Too many patients
		B	Lack of equipment/ supplies
		C	It is morally unacceptable because of religious considerations
		D	Lack of staff/ qualified personnel
		E	Facility does not have adequate space (e.g.theatre too small)
		X	Other (specify): ____________
302	In your opinion, how could treatment for abortion complications be improved at this facility?	A	Have a private room for postabortion care patients
		B	Improve/expand infrastructure
		C	Have more trained people available
		D	Have more lower level facilities equipped to provide PAC (postabortion care).
	**[Do not read]** **Circle all that apply.**	E	Have more commodities available (MVA equipment, antibiotics, blood, pain medication)
		F	Provide information on contraception and give methods
		G	Provide protocols for caring for postabortion patients
		H	Provide more incentives to staff (e.g. financial, refresher courses, material)
		I	Nothing is needed, current treatment is adequate
		X	Other (specify):
303	Currently in Malawi, the law only allows abortion to save a woman's life. Do you think the abortion laws in Malawi should be changed?	0	No
		1	Yes
		8	Don't know, no opinion, or not sure

304	Do you think the law should allow abortion in the following situations?<br><br>**[U = unsure, undecided, "it depends." U is a catch-all category for nuanced or ambivalent opinions.]**<br><br>**[Interviewer: Please read each reason and record response.]**		**Y**	**N**	**U**
		a. if the woman's physical health is at risk	1	0	8
		b. if the woman is mentally incapacitated	1	0	8
		m	1	0	8
		d. if the girl or woman is still in school	1	0	8
		e. if a girl is under age 18	1	0	8
		f. economic reasons (e.g. cannot care for the child)	1	0	8
		g. If the woman or girl is unmarried	1	0	8
		h. if pregnancy is from rape	1	0	8
		i. if pregnancy is from incest	1	0	8
		j. If pregnancy is from contraceptive failure	1	0	8
		k. if baby would be handicapped/foetal anomaly	1	0	8
		l. if the woman doesn't want the pregnancy	1	0	8
		x. other (specify)<br>____________________<br>____________________	1	0	8
305	Has your facility ever provided an abortion to save a woman's life (within the Malawian law)?	0 No **[Go to module 4]**<br>1 Yes			
306	How many times has this happened during the last 6 months, during the last 12 months, and during the last 5 years?	a. During the last 6 months [ ]<br>b. During the last 12 months [ ]<br>c. During the last 5 years [ ]			

Module 4: Abortion reporting

401	Do you keep or compile records on the number of women treated for abortion complications at this facility?	0<br>1	No **[End interview here.]**<br>Yes
402	How are these records kept?<br><br>**[If the facility keeps more than one type of records, circle the one that is the most complete and up-to-date.]**	1<br>2<br>3<br>4	Paper-based log book<br>Electronic, just in facility<br>Electronic, centralised in national or regional database<br>Other (specify):
403	Is there any written information about the number of women treated in this health facility (for abortion complications) that you can give me?<br><br>**[Interviewer: Record details at right and indicate if you are attaching additional information. It's best to obtain the information after the interview so as not to interrupt the interview right now. Obtain a photocopy of actual records, if available.]**	0<br>1	No **[End interview]**<br>Yes<br><br>**IF YES:**<br>Details:<br>**Number of women treated for complications of abortion:**<br>a. In the last month:<br>Outpatients<br>Inpatients<br>Total<br>b. In the last quarter:<br>Outpatients<br>Inpatients<br>Total<br>c. During the year 2014:<br>Outpatients<br>Inpatients<br>Total

END: Thank the respondent for their time.

(Interview: Remember to note time on title page)
