# Supplementary material for: Incidence of induced abortion in Malawi, 2015
Source: PLoS One. 2017 Apr 3;12(4):e0173639. doi: 10.1371/journal.pone.0173639 (PMC5378324; doi:10.1371/journal.pone.0173639)
Supplement: S3 File — (PDF) [file pone.0173639.s003.pdf]

Study of Abortion and Women's Health in Malawi

GUTTMACHER INSTITUTE

UNIVERSITY OF MALAWI

Knowledgeable Informant Survey on Condition of Abortion in Malawi

IDENTIFICATION

M1 **RESPONDENT IDENTIFICATION NUMBER** ________ | | | |

M2 **HEALTH ZONE** ________ | |

A. North B. Central-West C. Central-East D. Southwest E. Southeast

M3 **DISTRICT** ________ | | |

M4 **DATE OF INTERVIEW** | | | day | | | month |2|0|1| | Year

M5 **INTERVIEWER** ________ | | |

M6 **TIME STARTED:** | | |h | | |m

Complete rest of this page after the informed consent (and interview, if accepted) is complete.

M7 **TIME FINISHED:** | | |h | | |m

M8 **INTERVIEWER COMMENTARY ON RESPONDENT'S KNOWLEDGE OF ABORTION IN RURAL AREAS**

1	KNOWS VERY WELL
2	KNOWS MODERATELY WELL
3	HAS LIMITED INFORMATION
4	DOESN'T KNOW

M9 **INTERVIEW OUTCOME**

1	Completed
2	Refused - no incentive
3	Refused - any other reason
4	Incomplete
5	Not available for interview

Commentary:____

____

____

____

INTERVIEWER SIGNATURE (after interview): ____________________

QUALITY CONTROL SIGNATURE (after QC check): ____________________

DATA MANAGER SIGNATURE (after data entry): ____________________

Section 1: Basic Information

Number	Questions and Filters	Responses and Codes (Please circle neatly)	
101	Gender of respondent	1	Male
		2	Female
102	What is your primary profession?<br><br>**[Interviewer: DO NOT READ. Circle only one response. If respondent gives more than one profession, select the one that accounts for the greatest proportion of the respondent's time.]**	1	General physician
		2	Obstetrician/Gynecologist
		3	Nurse/Midwife
		4	Traditional healer
		5	Community health worker (HSA, CBD)
		6	Other health professional (*specify*): ____________
		7	Program manager/health administration
		8	Policy maker/policy advisor
		9	Activist/lawyer (e.g. in women's organization/issues)
		10	District Social Welfare Officer
		11	Community leader / youth leader
		12	Journalist / media person
		13	Researcher
		14	Lecturer/teacher
		96	Other *(specify):* ___________________________
103	In which sector do you work primarily?<br><br>**[Interviewer: If the respondent works in more than one sector, circle the category corresponding to the sector where he/she contributes the most time.]**	1	Private sector
		2	Public sector (including central or local government)
		3	Non-governmental organization (incl. FPAM & BLM)
		4	Christian Health Association of Malawi (CHAM)
		96	Other *(specify)* ______________________________
104	How long have you been working in your current profession? **[In completed years. If less than 12 months, enter "0" years]**	___ ___	Years
105	How old were you at your last birthday? **[In completed years]**	___ ___	Years
106	Do you **currently** work in an urban or rural area? Urban means town, city, or district BOMA. Rural is everything else.<br><br>**[Interviewer: If respondent works in different areas, circle the category corresponding to the area where he/she contributes the most time. If they spend about equal time in both, circle "both".]**	1	Urban area (includes peri-urban)
		2	Rural area
		3	Both
107	In your entire lifetime, for how long have you worked in a ***rural*** area?	___ ___	Years
		___ ___	Months

108	Please identify the different contexts in which you have encountered the issue of abortion as part of your professional experience?<br><br>**[Interviewer: Do not read response categories. Please circle all that apply.]**	A	In a public health facility framework
		B	In a private clinic framework
		C	Non-medical framework (research, policy-making, counseling, law) (Specify)____________________
		D	Through colleagues
		E	Other *(specify)* : ____________________

Section 2: Service provision

"In Malawi, very little is known about the provision of induced abortion—that is, steps taken or procedures done to end a pregnancy. Induced abortion may be done at any stage of pregnancy (that is, gestation), and it may be done by both trained and untrained providers in safe and unsafe conditions. We would like to have your opinion about several aspects of this area of reproductive behavior, to the extent that you are able to give an informed professional opinion. In this questionnaire, by abortion, we will be referring to induced abortion only.

The following questions are asked separately about women who live in urban and rural areas."

URBAN AREAS

201 "I will now read a list of methods; please indicate by saying yes or no, whether each method is or is not used in urban areas in Malawi to induce abortion, to the best of your knowledge?"
[Interviewer: Please read out each type of method and circle the appropriate response. Please circle all that apply from the list below, regardless of the type of practitioner who may use the method.]

DK=Do not know

**Type of Method [Read each method type]**	**Urban Areas**		
	**No**	**Yes**	**DK**
**a. Electric or manual vacuum aspiration (MVA)**	0	1	8
**b. Uterine evacuation with sharp curette (D&E or D&C)**	0	1	8
**c. Saline infusion**	0	1	8
**d. Oral introduction of drugs, solutions or other substances (i.e. through the mouth)**			
d1. Misoprostol alone, taken orally	0	1	8
d2. Contraceptive pills or other hormonal medications, taken orally (to induce abortion)	0	1	8
d3. Concoction, drink, tea, local herbs taken orally (except detergent)	0	1	8
d4. Detergent taken orally	0	1	8
d5. Overdose of pharmaceuticals (e.g. aspirin, chloroquine, metronidazole, DCN (doxycycline), La)	0	1	8
d6. Other *(specify)* ___________________________________	0	1	8
**e. Injection used for abortion**			
e1. Pitocin	0	1	8
e2. Any other injection (specify____)	0	1	8
**f. Vaginal introduction of drugs, solutions or other materials**			
f1. Misoprostol (Cytotec[R]) inserted vaginally	0	1	8
f2. Other hormonal medications, inserted vaginally	0	1	8
f3. Herbs or solutions (using any form of insertion into the vagina)	0	1	8
f4. Foreign objects (e.g. cassava stick, metal objects)	0	1	8
f5. Caustic agent (blue, permanganate)	0	1	8
f6. Catheter (with or without solution)	0	1	8
f7. Other *(specify)* ___________________________________	0	1	8
**g. Other means** (*specify any additional method(s) not listed above*)	0	1	8

"In your opinion, what is the most common method used to induce abortion by the following types of individuals:"

	**[Interviewer: Use the codes that correspond with the methods in Q201 to complete Q202-Q207. For example, if the respondent says "Drinking an herbal tea", enter d4. If type of provider does not exist in urban areas, write "97". If the respondent does not know the answer to the question, write "98".]**	**Urban areas**
202	Doctors or clinical officers in **urban** areas?	
203	Nurse/midwife or other trained provider who is not a doctor in **urban** areas?	
204	Traditional healers or TBAs in **urban areas**?	
205	Pharmacists or drug vendors in **urban** areas?	
206	Malawian women to self-induce abortion in **urban** areas?	
207	Other untrained person, such as friend, relative, or other in **urban** areas?	

"Each of the following questions asks you to consider two broad income groups – the urban poor and the relatively well-off (urban non-poor)."
[Interviewer: You can mention that there are not exact definitions for "poor" and "non-poor," but by "poor" we mean women with lower income levels.]

208 "I will mention the main types of people who perform induced abortions in Malawi. First thinking about urban areas, indicate whether, in your opinion, each type of provider is used rarely, sometimes or commonly by poor urban women seeking abortion."
[Interviewer: Read each type of individual and circle the respondent's answers for poor urban women in the first 4 columns. Mark all the respondent's answers relating to poor urban women, then ask the next question.]

"Now indicate whether, in your opinion, each type of individual is used rarely, sometimes or commonly by non-poor urban women."
[Interviewer: Read each type of individual and circle the respondent's answers for non-poor urban women in the last 4 columns.]

**[Read each type of person who may provide induced abortion]** R=rarely/never S=sometimes C=commonly NA=provider type doesn't exist in area	**1. Poor urban women**					**2. Non-poor urban women**			
	**R**	**S**	**C**	**NA**		**R**	**S**	**C**	**NA**
a. Doctor or clinical officer	1	2	3	4		1	2	3	4
b. Nurse/midwife or other trained provider who is not a doctor	1	2	3	4		1	2	3	4
c. Traditional healer or TBA	1	2	3	4		1	2	3	4
d. Pharmacist or drug vendor	1	2	3	4		1	2	3	4
e. Woman herself - self-induced	1	2	3	4		1	2	3	4
f. Other untrained person *(specify)* : ___________________________	1	2	3	4		1	2	3	4

Inteviewer: If the respondent indicates that a doctor is used sometimes or commonly (2 or 3) (whether by poor or non-poor women), circle the letter corresponding to the doctor. Do the same for all providers. Do not ask Q209 for providers whose letter has not been circled.

209 "In your opinion, what is the average amount that women living in urban areas pay for abortions, according to the type of provider they use?"
[Interviewer : Please verify using Q208 (=2 or 3) **and request a cost only for providers rated as used commonly or used sometimes. Amount should include the cost of services without transportation. If respondent does not know and will not guess, write "DK" in the response column. If respondent mentions a non-monetary amount, (i.e., trade of services for a goat) ask them to convert the cost of that item to the price in kwacha in their context.]**

**Type of people who may provide induced abortion**	**Average amount paid**
a. Doctor or clinical officer	
b. Nurse/midwife or other trained provider who is not a doctor	
c. Traditional healer or TBA	
d. Pharmacist or drug vendor	
e. Woman herself - self-induced	
f. Other untrained person *(specify)* : ___________________	

210 "In your opinion, what percentage of all induced abortions for **poor** women living in **urban areas** are being performed by each type of provider? Give an approximate percentage (all providers sum to 100%)."
[Interviewer: Please read each type of provider. Confirm that all providers sum to 100%. If they do not, probe for a correction, and adjust the answers below. Allow the respondent to fully answer the question for poor women, then ask the following question:]

"Now, in your opinion, what percentage of all induced abortions for **non-poor** women living in **urban areas** are being performed by each type of provider? Give an approximate percentage (all providers sum to 100%)."

**Type of Provider**	**Percentage**	
	**1. Poor urban women**	**2. Non-poor urban women**
a. Doctor or clinical officer	%	%
b. Nurse/midwife or other trained provider who is not a doctor	%	%
c.Traditional healer or TBA	%	%
d. Pharmacist or drug vendor	%	%
e. Woman herself - self-induced	%	%
f. Other untrained person (specify):____________________	%	%
**TOTAL**	**100 %**	**100 %**

RURAL AREAS

"Now I am going to ask you the same questions as above, but this time for rural areas."

211 "I will now read a list of methods; please indicate by saying yes or no, whether each method is or is not used in rural areas in Malawi to induce abortion, to the best of your knowledge?"
[Interviewer: Please read out each type of method and circle the appropriate response. Please circle all that apply from the list below, regardless of the type of practitioner who may use the method.]

DK=Do not know

**Type of Method [Read each method type]**	**Rural Areas**		
	**No**	**Yes**	**DK**
**a. Electric or manual vacuum aspiration (MVA)**	0	1	8
**b. Uterine evacuation with sharp curette (D&E or D&C)**	0	1	8
**c. Saline infusion**	0	1	8
**d. Oral introduction of drugs, solutions or other substances (i.e. through the mouth)**			
d1. Misoprostol alone, taken orally	0	1	8
d2. Contraceptive pills or other hormonal medications, taken orally (to induce abortion)	0	1	8
d3. Concoction, drink, tea, local herbs taken orally (except detergent)	0	1	8
d4. Detergent taken orally	0	1	8
d5. Overdose of pharmaceuticals (e.g. aspirin, chloroquine, metronidazole, DCN (doxycycline), La)	0	1	8
d6. Other *(specify)* ________________________________	0	1	8
**e. Injection used for abortion**			
e1. Pitocin	0	1	8
e2. Any other injection (specify__________________________________)	0	1	8
**f. Vaginal introduction of drugs, solutions or other materials**			
f1. Misoprostol (Cytotec[R]) inserted vaginally	0	1	8
f2. Other hormonal medications, inserted vaginally	0	1	8
f3. Herbs or solutions (using any form of insertion into the vagina)	0	1	8
f4. Foreign objects (e.g. cassava stick, metal objects)	0	1	8
f5. Caustic agent (blue, permanganate)	0	1	8
f6. Catheter (with or without solution)	0	1	8
f7. Other *(specify)* ________________________________	0	1	8
**g. Other means** (*specify any additional method(s) not listed above)*	0	1	8

"In your opinion, what is the most common method used to induce abortion by the following practitioner:"

[Interviewer: Use the codes that correspond with the methods in Q211 to complete Q212-Q217. For example, if the respondent says "Drinking an herbal tea", enter d4. If type of provider does not exist in rural areas, write "97". If respondent does not know, write "98".]

		Rural areas		
212	Doctors or clinical officers in **rural** areas?			
213	Nurse/midwife or other trained provider who is not a doctor in **rural** areas?			
214	Traditional healer or TBA in **rural areas**?			
215	Pharmacists or drug vendors in **rural** areas?			
216	Malawian women to self-induce abortion in **rural** areas?			
217	Other untrained person (specify______________________________) in **rural** areas?			

"Each of the following questions asks you to consider two broad income groups – the rural poor and the relatively well-off (rural non-poor)."

[Interviewer: You can mention that there are not exact definitions for "poor" and "non-poor," but by "poor" we mean women with lower income levels.]

218 "Once again, I will mention the main types of people who perform induced abortions in Malawi. Considering rural areas, indicate whether, in your opinion, each type of provider is used rarely, sometimes or commonly by poor rural women seeking abortion."

[Interviewer: Please read each type of provider and circle the respondent's answers for poor rural women in the first 4 columns. Mark all the respondent's answers relating to poor rural women, then ask the next question.]

"Now indicate whether, in your opinion, each type of provider is used rarely, sometimes or commonly by non-poor rural women."

**Type of Provider** **[Read each type]** (R=rarely/never, S=sometimes, C=commonly, NA=provider type doesn't exist in area)	**1. Poor rural women**					**2. Non-poor rural women**			
	**R**	**S**	**C**	**NA**		**R**	**S**	**C**	**NA**
a. Doctor or clinical officer	1	2	3	4		1	2	3	4
b. Nurse/midwife or other trained provider who is not a doctor	1	2	3	4		1	2	3	4
c. Traditional healer or TBA	1	2	3	4		1	2	3	4
d. Pharmacist or drug vendor	1	2	3	4		1	2	3	4
e. Woman herself - self-induced	1	2	3	4		1	2	3	4
x. Other untrained person *(specify)* : ________________________________	1	2	3	4		1	2	3	4

Inteviewer : If the respondent indicates that a doctor is used sometimes or often (2 or 3) (whether by poor or non-poor women), circle the letter corresponding to the doctor. Do the same for all providers. Do not ask Q219 for providers whose letter has not been circled.

219 "In your opinion, what is the average amount that women living in **rural areas** pay for abortions by type of provider they use?"

[Interviewer : Please verify using Q218 (=2 or 3) and request an average cost only for tproviders rated as used commonly or used sometimes. Amount should include the cost of services without transportation. If respondent does not know and will not guess, write "DK" in the response column. If respondent mentions a non-monetary amount, (i.e., trade of services for a goat) ask them to convert the cost of that item to the price in kwacha in their context.]

**Type of Provider**	**Average amount paid**
a. Doctor or clinical officer	
b. Nurse/midwife or other trained provider who is not a doctor	
c. Traditional healer or TBA	
d. Pharmacist or drug vendor	
e. Woman herself - self-induced	
x. Other untrained person *(specify)* : ____________________	

220 "In your opinion, what percentage of all induced abortions for **poor** women living in **rural areas** are being performed by each type of provider? Give an approximate percentage (all providers sum to 100%)."

[Interviewer: Please read each type of provider. Confirm that all providers sum to 100%. If they do not, probe for a correction, and adjust the answers below. Allow the respondent to fully answer the question for poor women living in rural areas, then ask the following question:]

"Now, in your opinion, what percentage of all induced abortions for **non-poor** women living in **rural areas** are being performed by each type of provider?"

Type of Provider	Percentage							
	1. Poor rural women				2. Non-poor rural women			
a. Doctor or clinical officer				%				%
b. Nurse/midwife or other trained provider who is not a doctor				%				%
c.Traditional healer or TBA				%				%
d. Pharmacist or drug vendor				%				%
e. Woman herself - self-induced				%				%
f. Other untrained person (specify):____________________				%				%
**TOTAL**			**100**	**%**			**100**	**%**

"Now I would like to ask you a few questions about what you think are the barriers preventing some women (either urban or rural), from using post-abortion care services."

221 What do you think are the reasons for not using or delaying seeking postabortion care services at a facility?

[Interviewer: Tick all answers that the respondent spontaneously mentions.]

A	Financial cost of services (not transport)	
B	Distance/ transportation	
C	Stigma	
D	Inadequate training of providers	
E	Inadequate equipment at facility	
F	Under-staffing at facility	
G	Hostile/unfriendly provider attitudes	
H	Lack of information about services	
I	Husband/family objections	
J	Fear of prosecution/arrest	
K	To avoid being compelled to continue the pregnancy against their will	
L	Religious reasons	
X	Other (specify):	

222 "What do you think would help reduce these barriers that prevent women from using postabortion care services at a facility?"

[Interviewer: Tick all answers that the respondent spontaneously mentions.]

Are there any other important strategies that can be used to eliminate the barriers that prevent women from using postabortion care services?]

A	Reduce financial cost	
B	Increase number of providers/clinics	
C	Increase or improve clinic hours	
D	Ensure confidential services	
E	Improve provider training	
F	Provide more or better equipment	
G	Increase staffing	
H	Ensure non-judgemental provider attitudes	
I	Provide information to women on post-abortion care services	
J	Change law on abortion	
K	Attend to PAC patients promptly	
L	Train and equip non-facility based providers	
X	Other (specify):	

Section 3: Abortion Complications

"Now, I will talk to you about abortion complications from induced abortion, meaning an abortion that is provoked. As you know, complications that result from induced abortion range from mild to very severe. When we speak of abortion complications, we are referring to those consequences **that are severe enough to need treatment** in a health facility**, whether or not the woman actually obtains health care. Complications due to abortion, as defined here, include not only the extremely serious cases such as those with infection or trauma to the uterus, but also those cases which are termed "incomplete abortions," which are usually identified by heavy bleeding, and which present a somewhat less severe health risk to the woman, but which, nevertheless, require hospitalization. In answering the following set of questions concerning abortion complications, please keep this definition in mind."**

301 "Which complications resulting from induced abortion are common in Malawi?"

[Interviewer: Please circle all that apply. Do not prompt.]

A	"Incomplete" abortion
B	Excessive loss of blood (hemorrhage)
C	Infection of uterus and/or surrounding areas (sepsis or "septic abortion")
D	Septic shock
E	Injury to the vagina/cervix
F	Injury to the uterus (i.e. perforation)
G	Injury to the internal organs
H	Pain in lower abdomen
I	Infertility
J	Death
X	Other *(specify)* :_________________________ ____

URBAN AREAS

"First, I will ask you questions about urban women only."

302 "Think about **poor women** in **urban areas**: out of 10 poor urban women who have an induced abortion performed by each type of provider that I will mention, how many would experience a complication that needs medical treatment, whether or not they actually receive treatment? There is no need for these numbers to sum to 10 when combined."

[Interviewer: Ask for each type of provider separately; insert a number in each column, even though it might be "0." If a particular type of provider does not exist in urban areas, code "98". Allow the respondent to fully answer this question, then ask the following question:]

"Now what about **non-poor women** in **urban areas:** out of 10 *non-poor* women having procured abortions from each of the providers below, how many would have a complication requiring medical treatment, regardless of whether they receive treatment?"

Type of provider	Number of women out of 10 with medical complications	
	**1. Poor urban women**	**2. Non-poor urban women**
a. Doctor or clinical officer (any place)		
b. Nurse, midwife, or medical assistant		
c. Traditional healer or TBA		
d. Pharmacist or drug vendor		
e. Woman - self-induced		
f. Other untrained person (specify): ______________ ____		

303	"Think about **poor women** in **urban areas**: out of 10 poor urban women who experience **a medical complication** due to an induced abortion, how many do you think would be treated by a trained person in a health facility?"<br><br>**[Interviewer: Allow the respondent to fully answer this question, then ask the following question:]**<br><br>"What would the number be for **non-poor women** living in an **urban area**?"	**Number of women out of 10 treated in an urban health facility**<br>**a. Poor urban women** ☐	**b. Non- poor urban women** ☐
**"Not all induced abortions in Malawi are unsafe. There are instances in which a woman can obtain an abortion from a trained provider who uses a safe abortion technique and appropriate instruments in a hygenic enviroment."**			
304	"Thinking first of **(a) urban poor** women: to the best of your knowledge, what percent of induced abortions would you say are safe according to this criteria?"<br>"Thinking now of **(b) urban non-poor** women; to the best of your knowledge, what percent of induced abortions would you say are safe according to this criteria?"	**a. Poor urban women**<br>☐☐ %	**b. Non-poor urban women**<br>☐☐ %
**"Now I have some questions about urban women who seek care for other services. First, please think about urban women who have miscarriages, also called spontaneous abortions, where a pregnancy ends by itself without anyone doing anything to end it."**			
305	a. "Of all urban women who miscarry a pregnancy before 3 months gestation (**6-12 weeks)** gestation, what percentage seek treatment in a health facility?"<br><br>b. "And, of all urban women who miscarry a pregnancy at over 3 months up to 5 months (**13-21 weeks)** gestation, what percentage seek treatment in a health facility?"	**a. 3 months gestation**<br>**(6-12 weeks)**<br>☐☐ %	**b. over 3 mos up to 5 mos**<br>**(13-21 weeks)**<br>☐☐ %
306	"Now, please consider **urban women** giving birth to a baby: Out of all women in urban areas giving birth, what percentage deliver their baby in a health facility?"	☐☐ %	

RURAL AREAS

Now, I will ask you questions about rural women only.

307 "Now think about **poor women** in **rural areas**: out of 10 **poor urban** women who have an induced abortion performed by each type of provider that I will mention, how many would experience a medical complication that should receive medical treatment? There is no need for these numbers to sum to 10 when combined."

[Interviewer: Ask for each type of provider separately; insert a number in each column, even though it might be "0." If a particular type of provider does not exist in rural areas, code "98". Allow the respondent to fully answer this question, then ask the following question:]

"Now what about **non-poor women** in **rural areas**: out of 10 non-poor women having procured abortions from each of the providers below, how many would have a complication requiring medical treatment?"

Type of provider	Number of women out of 10 with medical complications	
	**1. Poor rural women**	**2. Non-poor rural women**
a. Doctor or clinical officer (any place)		
b. Nurse/midwife, or other trained provider who is not a doctor		
c. Traditional healer or TBA		
d. Pharmacist or drug vendor		
e. Woman - self-induced		
f. Other untrained person (specify): _____________ ____		

308 "Now think about **poor women** in **rural areas**: out of 10 poor rural women who experience **a medical complication** due to an induced abortion, how many do you think would be treated by a trained person in a health facility?"

[Interviewer : Allow the respondent to fully answer this question, then ask the following question:]

"What would the number be for **non-poor women** living in a **rural area**?"

Number of women out of 10 treated in an rural health facility	
**a. Poor rural women**	**b. Non- poor rural women**

"Not all induced abortions in Malawi are unsafe. There are instances in which a woman can obtain an abortion from a trained provider who uses a safe abortion technique and appropriate instruments in a hygenic enviroment."

309 "Thinking first of **(a) rural poor** women: to the best of your knowledge, what percent of induced abortions would you say are safe according to this criteria?"

"Thinking now of **(b) rural non-poor** women, to the best of your knowledge, what percent of induced abortions would you say are safe according to this criteria?"

**a. Poor rural women**	**b. Non-poor rural women**
__ __ %	__ __ %

"I'm going to ask again about rural women seeking care for other services. Again, I will start by asking about miscarriages, also called spontaneous abortions, where a pregnancy ends by itself without anyone doing anything to end it."

310 a. "Of all rural women who miscarry a pregnancy before **3 months gestation** (6-12 weeks), what percentage seek treatment in a health facility?"

b. "And, of all rural women who miscarry a pregnancy at **over 3 months up to 5 months** (13-21 weeks) gestation, what percentage seek treatment in a health facility?"

**a. 3 months gestation (6-12 weeks)**	**b. over 3 mos up to 5 mos (13-21 weeks)**
__ __ %	__ __ %

311 "Now, please consider **rural women** giving birth to a baby: Out of all women in rural areas giving birth, what percentage deliver their baby in a health facility?"

__ __ %

Section 4: Use of misoprostol

Misoprostol (or Cytotec) is sometimes used in Malawi for hemorrhage after childbirth and postabortion care. There is the possibility that it is also being used for inducing abortion. I would like to ask your perceptions about whether and how much misoprostol is used for induced abortion in Malawi.

401	As far as you are aware, is misoprostol currently being used by women in Malawi to induce abortions?	0<br>1<br>8	No **[SKIP TO SECTION 5]**<br>Yes<br>Don't know **[SKIP TO SECTION 5]**
402	In what year do you think misoprostol first started being used in Malawi to induce abortion?<br><br>**[Interviewer: Write answer in complete years only]**		
403	As far as you are aware, is misoprostol used for abortion in **urban** areas of Malawi commonly, sometimes, rarely, or never?	1<br>2<br>3<br>8	Commonly<br>Sometimes<br>Rarely/never<br>Don't know
404	As far as you are aware, is misoprostol used for abortion in **rural** areas of Malawi commonly, sometimes, rarely, or never?	1<br>2<br>3<br>8	Commonly<br>Sometimes<br>Rarely/never<br>Don't know

405	As far as you are aware, at what types of facilities or places do you think misoprostol is provided or sold?<br><br>**[Interviewer: Do not read list. Multiple responses allowed. Please circle all that apply.]**	A	Doctors' private office
		B	Nurses' or clinical officers' private office
		C	Health facilities (public)
		D	Health facilities (private)
		E	NGOs (including BLM and FPAM)
		F	CHAM facilities
		G	Traditional healers
		H	Pharmacists or drug vendors
		I	Markets or street vendors
		X	Other (specify): ____________________
		Z	Don´t know

Interviewer: Check question 403. If 403 is 3 (rarely or never) skip to 407.

406 Thinking first about women in urban areas, what percent of **urban poor women** obtaining abortions from each type of provider do you think use misoprostol to induce an abortion?

What about **non-poor women** living in **urban areas**? Thinking about all **non-poor** women living in **urban** areas who obtain abortions from doctors, what percent of them use misoprostol for their abortion?

	**% of women obtaining abortions from each provider who use misoprostol**	
	**a. Urban poor women**	**b. Urban non-poor women**
a. Doctor or clinical officer (any place)		
b. Nurse, midwife, or medical assistant		
c. Traditional provider or TBA		
d. Pharmacist or drug vendor		
e. Woman - self-induced		
f. Other untrained person (specify: ____________________ ____________________)		

Interviewer: Check question 404. If 404 is 3 (rarely or never) skip to 408.

407 Now I have the same question about women living in **rural** areas. What percent of **rural poor women** do you think use misoprostol to induce an abortion?

What about **non-poor women** living in **rural areas**?

	**% of women obtaining abortions from each provider who use misoprostol**	
	**a. Rural poor women**	**b. Rural non poor women**
a. Doctor or clinical officer (any place)		
b. Nurse, midwife, or medical assistant		
c. Traditional provider or TBA		
d. Pharmacist or drug vendor		
e. Woman - self-induced		
f. Other untrained person (specify: ____________________ ____________________)		

Interviewer: Check question 403. If 403 is 3 (rarely or never) skip to 411.

URBAN WOMEN

408 Think about poor women in urban areas who use misoprostol to induce an abortion: out of 10 poor, urban women using misoprosto from each type of provider that I will mention, how many would seek medical care because of misoprostol-induced bleeding that would have resolved itself on its own (i.e. without any health facility intervention)?

What about non-poor women in urban areas?

	Number of women out of 10 *without* complications who seek treatment	
	**a. Urban poor women**	**b. Urban non-poor women**
a. Doctor or clinical officer (any place)		
b. Nurse, midwife, or medical assistant		
c. Traditional provider or TBA		
d. Pharmacist or drug vendor		
e. Woman - self-induced		
f. Other untrained person (specify: ___________________ ____)		

409 Think about poor women in urban areas who use misoprostol to induce an abortion: out of 10 poor, urban women using misoprosto from each type of provider that I will mention, how many would experience a complication that needs medical treatment, whether or not they actually receive treatment? Please do not include the number of women experiencing the expected bleeding associated with misoprostol use (whom we discussed just previously).

What about non-poor women in urban areas?

	Number of women out of 10 with complications	
	**a. Urban poor women**	**b. Urban non-poor women**
a. Doctor or clinical officer (any place)		
b. Nurse, midwife, or medical assistant		
c. Traditional provider or TBA		
d. Pharmacist or drug vendor		
e. Woman - self-induced		
f. Other untrained person (specify: ___________________ ____)		

410 Think about **poor women** in urban areas: out of 10 poor, urban women who experience **a medical complication** from using misoprostol, how many do you think would be treated by a trained person in a health facility?

[Interviewer : Allow the respondent to fully answer this question, then ask the following question:]

What would the number be for **non-poor women** (living in urban areas)?

Number of women out of 10 treated in a health facility for complications from misoprostol abortion	
**a. Urban poor women**	**b. Urban non-poor women**

Interviewer: Check question 404. If 404 is 3 (rarely or never) skip to 414.

RURAL WOMEN

411 Think about poor women in rural areas who use misoprostol to induce an abortion: out of 10 poor, rural women using misoprostol from each type of provider that I will mention, how many would seek medical care because of misoprostol-induced bleeding that would have resolved itself on its own (i.e. without any health facility intervention)?

What about non-poor women in rural areas?

	**Number of women out of 10 *without* complications who seek treatment**	
	**a. Rural poor women**	**b. Rural non-poor women**
a. Doctor or clinical officer (any place)		
b. Nurse, midwife, or medical assistant		
c. Traditional provider or TBA		
d. Pharmacist or drug vendor		
e. Woman - self-induced		
f. Other untrained person (specify: ___________________ ____)		

412 Think about poor women in rural areas who use misoprostol to induce an abortion: out of 10 poor, rural women using misoprostol from each type of provider that I will mention, how many would experience a complication that needs medical treatment, whether or not they actually receive treatment? Please do not include the number of women experiencing the expected bleeding associated with misoprostol use (whom we discussed just previously).

What about non-poor women in rural areas?

	**Number of women out of 10 with complications**	
	**a. Rural poor women**	**b. Rural non-poor women**
a. Doctor or clinical officer (any place)		
b. Nurse, midwife, or medical assistant		
c. Traditional provider or TBA		
d. Pharmacist or drug vendor		
e. Woman - self-induced		
f. Other untrained person (specify: ___________________ ____)		

413		**Number of women out of 10 treated in a health facility for complications from misoprostol abortion**	
		**a. Rural poor women**	**b. Rural non-poor women**
	Think about women in rural areas: out of 10 **poor, rural** women who experience a medical complication from using misoprostol, how many do you think would be treated by a trained person in a health facility?<br><br>[Interviewer : Allow the respondent to fully answer this question, then ask the following question:]<br><br>What would the number be for **non-poor** women (living in rural areas)?		

414	The preceeding sections included questions that required you to give your opinion on concepts that are not easily measured. On a scale of 1 to 10, with 1 being "not at all sure" and 10 being "very sure", what is your degree of certainty that the answers you've given reflect the real situation encountered in Malawi for abortions in **urban areas**?	**Misoprostol use in urban areas**<br>10 Very sure<br>9<br>8<br>7<br>6<br>5<br>4<br>3<br>2<br>1 Not at all sure
415	On a scale of 1 to 10, with 1 being "not at all sure" and 10 being "very sure", what is your degree of certainty that the answers you've given reflect the real situation encountered in Malawi for abortion in **rural areas**?	**Misoprostol use in rural areas**<br>10 Very sure<br>9<br>8<br>7<br>6<br>5<br>4<br>3<br>2<br>1 Not at all sure

Section 5: General

501	"Please mention any suggestions or recommendations that you feel could be used in Malawi to lower the level of unintended pregnancies."<br><br>**[Interviewer: Do not read. Multiple responses are allowed.]**		
		A	Increase availability of family planning services
		B	Improve the quality of contraceptive counseling and services
		C	Improve the provision of contraceptive counselling post delivery and/or post-abortion
		D	Implement campaigns to educate public about family planning programs and increase support
		E	Improve provision of sex education in schools, universities and communities
		X	Other (*specify*):
502	"Please mention any suggestions or recommendations that you feel could be used in Malawi to reduce the number of unsafe abortions and their consequences for women's health."<br><br>**[Interviewer: Do not read. Multiple responses are allowed.]**		
		A	Improve the coverage and quality of post abortion care services
		B	Publicize the health risk involved in unsafe abortion
		C	Change the legal status of abortion
		D	Improve information and access to contraception
		E	Improve access to safe abortion services
		F	Encourage male involvement in family planning
		G	Discourage premarital sex
		H	Discourage abortion
		I	No opinion
		X	Other (*specify*):
		Z	Don't know
503	"Currently in Malawi, the law only allows abortion to save a woman's life. Do you think the abortion laws in Malawi should be changed?"	0	No
		1	Yes
		8	No opinion/Don't know

504 "Do you think abortion should be allowed in the following situations?"

Situations	No	Yes	Don't know
a. If the woman's health is at risk	0	1	8
b. If the woman is mentally incapacitated	0	1	8
c. If the woman's mental health is at risk	0	1	8
d. If the girl is under 18	0	1	8
e. If the girl or woman is still in school	0	1	8
f. Economic reasons (e.g. cannot care for the child)	0	1	8
g. If the woman or girl is unmarried	0	1	8
h. If the pregnancy is from rape	0	1	8
i. If the pregnancy is from incest	0	1	8
j. If the pregnancy is from contraceptive failure	0	1	8
k. If the baby would be handicapped/foetal anomaly	0	1	8
l. If the woman doesn't want the pregnancy	0	1	8
x. Other reasons (please specify:)	0	1	8

[Interviewer : Please give your commentary on the respondent's knowledge of rural areas on the cover page. Remember to note timing of the end of interview on title page.]

"Thank you very much for taking the time to share your knowledge with me today. If you are interested in seeing the final publication based on this survey after it is completed (likely in 2017), you may contact the Principal Investigator.
